# Framework for standardized genetic testing recommendations for chronic kidney disease in Ontario

**DOI:** 10.1016/j.gimo.2025.103442

**Published:** 2025-07-05

**Authors:** Angela Du, Kaitlyn Lemay, Amit Bagga, Priya T. Bhola, Pierre Antoine Brown, Samantha Colaiacovo, George S. Charames, Mathieu Lemaire, Matthew B. Lanktree, Laila Schenkel, Luis G. Peña, Samantha Riddell, Nicholas Watkins, Ted Young, Wilson Yu, Kathleen Bell, Raymond H. Kim, Dervla M. Connaughton, Ontario Health Provincial Genetics Program Renal Genetics Expert Group, Andrea Guerin, Angela Du, Kaitlyn Lemay, Kathleen Bell, Muna Aden, Raymond Kim, Wilson Yu

**Affiliations:** 1 Provincial Genetics Program, Ontario Health, Toronto, ON, Canada; 2 Windsor Regional Hospital, Windsor, ON, Canada; 3 Children’s Hospital of Eastern Ontario, Ottawa, ON, Canada; 4 Division of Nephrology, Department of Medicine, University of Ottawa and The Ottawa Hospital, Ottawa, ON, Canada; 5 Department of Clinical Genetics, Victoria Hospital, London Health Sciences Centre, London, ON, Canada; 6 Department of Molecular Genetics, University of Toronto, Toronto, Canada; 7 Department of Lab Medicine and Pathobiology, University of Toronto, Toronto, ON, Canada; 8 Lunenfeld-Tanenbaum Research Institute, Sinai Health System, Toronto, ON, Canada; 9 Hospital for Sick Children, Toronto, ON, Canada; 10 Departments of Medicine and Health Research Methodology, Evidence, and Impact, McMaster University, Hamilton, ON, Canada; 11 Division of Nephrology, St Joseph's Healthcare Hamilton, Population Health Research Institute, Hamilton, ON, Canada; 12 Pathology and Laboratory Medicine, London Health Sciences Centre and the University of Western Ontario, London, ON, Canada; 13 Health Sciences North, Sudbury, ON, Canada; 14 Mount Sinai Hospital, Toronto, ON, Canada; 15 Division of Medical Oncology and Hematology, Princess Margaret Cancer Centre, Sinai Health System, Toronto, ON, Canada; 16 Schulich School of Medicine & Dentistry, Western University, London, ON, Canada; 17 Division of Nephrology, Department of Medicine, University Hospital, London Health Sciences Centre, London, ON, Canada

**Keywords:** Chronic kidney disease, Genetics testing, Genomic medicine, Implementation, Nephrogenetics

## Abstract

**Purpose:**

Genetic causes account for 10% to 20% of adult and 30% to 50% of pediatric chronic kidney disease (CKD). Patients with genetic CKD have a higher risk of progression to kidney failure. More than 500 genes are implicated in kidney disease; yet, Ontario’s existing gene panel options includes fewer than 45 genes. Despite growing evidence for genetic testing in CKD care, testing is not systematically integrated into the diagnostic pathway. Standardized testing and clear eligibility criteria are needed to improve diagnosis, care, and outcomes.

**Methods:**

In 2023, Ontario Health’s Provincial Genetics Program convened a Renal Genetics Expert Group to develop standardized genetic testing criteria and evidence-based multigene panels for CKD. This initiative aims to support equitable access to high-quality genetic services and improve clinical outcomes through early, accurate diagnoses.

**Results:**

An environmental scan of provincial, national, and international guidelines informed the development of a testing framework. Literature review and expert consensus guided the creation of eligibility criteria and panel content. Input from nephrologists, geneticists, genetic counsellors, and patients was incorporated throughout the process.

**Conclusion:**

Standardized recommendations for genetic testing in CKD promote consistent, equitable access to diagnostics across Ontario. Careful curation of multigene panels that align with current knowledge of gene-disease associations and patient phenotypes, can help streamline testing. Integration of this framework into clinical care will strengthen collaboration between nephrology and genetics, facilitate earlier diagnosis, and support personalized management, ultimately improving outcomes for individuals with CKD.

## Introduction

In Canada, there are at least 4 million people with chronic kidney disease (CKD), consistent with an estimated global prevalence of 11% to 13%.^1^, ^2^, ^3^ A 2020 Canadian study found that over 50,000 individuals in Canada have kidney failure, with the highest prevalence in Ontario.^4^ To date, hundreds of monogenic causes of CKD have been identified.^5^, ^6^, ^7^, ^8^ Although individually rare, genetic causes of kidney disease account for 10% to 20% of CKD cases in adults,^8^^,^^9^ with a higher prevalence in children and in individuals with additional risk factors.^6^^,^^10^^,^^11^ For example, those with positive family history of CKD, extrarenal features, or specific subtypes of CKD are more likely to have a genetic form of kidney disease.^5^^,^^6^^,^^12^^,^^13^ Recent evidence demonstrates that instituting a workflow, based on well-defined clinical criteria for genetic testing, leads to high diagnostic rates following genetic assessment (>50%).^14^, ^15^, ^16^, ^17^

Data also shows that confirming a diagnosis of genetic kidney disease leads to a meaningful change in management for 90% of cases, including changes in treatment plans, referrals to genetic counselling, recommendations for family member referrals to genetic testing and informing discussions around family planning.^9^ Despite these benefits, genetic testing is not yet a routine part of the diagnostic pathway for CKD and too often performed late in the process.^10^^,^^18^ Along with enhancing clinical utility, genetic testing can be cost-effective, especially when done early in the diagnostic pathway.^16^^,^^19^^,^^20^ The cost of CKD to the Canadian health care system remains high, estimated at $40 billion annually, much of which is driven by the progression to kidney failure.^2^^,^^21^ Early detection is important, considering that patients with genetic kidney disease are at a higher risk of progression to kidney failure compared with those with nongenetic forms of CKD.^22^ In addition, an Australian study has shown that early comprehensive genetic testing done in patients with suspected CKD results in cost savings.^16^^,^^19^

The Kidney Disease Improving Global Outcomes (KDIGO) Guidelines recommend genetic testing in the diagnostic pathway for all CKD patients with suspected genetic kidney disease, with no upper age limit for testing.^12^ KDIGO also highlighted the utility in patients with CKD of unknown etiology, particularly in advanced disease in which a kidney biopsy would be uninformative.^23^ Confirming a genetic diagnosis can have added benefits beyond the index patient, by facilitating early identification of at-risk family members with cascade screening thereby reducing the diagnostic odyssey for subsequent family members.^10^ Therefore, integration of genetic testing should be considered in all cases in which the diagnosis of genetic kidney disease can support family planning, reduce costs, reduce time to diagnosis, potentially negate the need for unnecessary or invasive diagnostic procedures, and/or impact treatment.^7^^,^^23^, ^24^, ^25^, ^26^

In Canada, the Province of Ontario’s Ministry of Health (MOH) has developed the Provincial Genetics Program (PGP) at Ontario Health. The PGP is mandated by the MOH to “ensure the successful implementation of genetic testing and establishment of a comprehensive provincial program for all genetics with robust provincial oversight to deliver these services to drive better outcomes for Ontarians and improved value.”^27^ The PGP was launched in April 2021, and kidney genetics was identified as a priority domain for development in Ontario, resulting in the formation of the Renal Genetics Expert Group in April 2023 (Supplemental Figure 1). The role of the expert group is to develop evidence-based guidance for providing genetic diagnostics and counselling services. Ontario Health’s mandate to oversee genetic testing aims to establish standardized guidelines that ensure consistent eligibility criteria, equitable access, and evidence-based clinical practices across Ontario. Herein, we report the PGP Renal Genetics Expert Group’s recommendations. These recommendations primarily relate to the eligibility for genetic assessment and testing for kidney disease, focusing on genetic testing in affected individuals, clinical implementation considerations, and evidence-based multigene panels. This guidance document includes recommendations on which patients with CKD should be considered for genetic testing and what testing strategy should be used.

## Materials and Methods

### Environmental scan

An environmental scan was conducted to evaluate the current landscape of kidney genetics in Ontario, Canada, and internationally. The objectives were to identify existing genetic testing guidelines and practices for CKD and to assess gaps in the availability and composition of genetic testing panels in Ontario and evaluate their alignment with national and international standards. The scope of the scan covered guidelines and practices at provincial, national, and international levels.^28^, ^29^, ^30^ Multiple data collection sources were used as outlined in Table 1.

**Table 1:** Data source for development of guidelines and eligibility criteria

Source Type	Details
Scientific literature	Databases: PubMed and Embase
Clinical guidelines	National guidelines (eg, Canadian, Dutch, Australian, United Kingdom), international (eg, KDIGO), and professional organizations’ documents.
Governmental and health organization reports	Reports from entities such as Ontario Health or the Ministry of Health.
Gray literature	Includes white papers, policy briefs, and non-peer-reviewed reports.
Expert input	Conduct interviews or consultations with clinicians, geneticists, and policymakers.

*KDIGO*, Kidney Disease Improving Global Outcomes.

Specific areas of analysis included existing genetic testing guidelines in CKD, gene coverage in genetic testing panels in Ontario, utilization of out-of-province (ie, outside Ontario) and out-of-country gene testing (ie, outside Canada), application processes to MOH for genetic testing in patients with CKD and access to genome-wide sequencing (GWS), which includes both exome and genome sequencing, through Genome-wide Sequencing Ontario platform.^31^

### Priority setting

The 14-member Renal Genetic Expert Group comprises clinical and molecular geneticists, adult and pediatric nephrologists, subspecialists in kidney transplantation, genetic counsellors, laboratory specialists, bioinformatic and genomic analyses experts, and health policy and implementation scientists. The group conducted an initial priority-setting exercise to identify key gaps in genetic testing for CKD in our jurisdiction. To achieve this, a structured consensus-building approach incorporating surveys, group discussions, and voting was used. Prioritization criteria included clinical utility, diagnostic yield, cost-effectiveness, and equity of access to genetic testing. Gaps were ranked, and recommendations were tailored to improve access to testing and address specific needs, such as genetic testing for underrepresented CKD subtypes.

### Development of guidance

All sources of literature were included up to December 31, 2024. Recommendations were developed and refined by the expert group based on a review of the literature and expert consensus. The consensus process included a literature review as outlined below. Search strategies were used in PubMed and Embase, ensuring a rigorous evidence base (Supplemental Table 1). Expert group meetings were held weekly by the clinical lead and core team, and quarterly meetings with the 14-member expert group, to facilitate discussion of key topics and iterative refinement of recommendations. In some cases, subworking groups were convened to address specific topics, including cystic kidney disease and complement-mediated kidney disease. All expert group members were asked to vote on each specific eligibility criteria for genetic testing and the inclusion/exclusion of genes in each disease-specific panel. Feedback provided by expert group members through voting was reflected in revised eligibility criteria statements, which were shared with the group for final approval (Supplemental Figure 2). The review was enhanced and validated by external review from experts both within Canada and internationally (Supplemental Table 2). Reviewers were asked to evaluate interim drafts and provide feedback, which were incorporated into final recommendations. Feedback was also provided by the Equity Lead at the PGP, the Primary Care Genetics Reference Table, and the Patient and Care Partners Genetics Reference Table. The intended audience for these guidance documents includes clinical and molecular geneticists, genetic counsellors, nephrologists, urologists, transplant physicians, and laboratory specialists. It excludes polygenic risk scores, unvalidated risk alleles (except *APOL1*), and conditions covered by other expert groups (eg, monogenic diabetes, aldosteronism, and kidney cancer genetics) and testing pertaining to prenatal assessments.

### Data extraction and analysis

As previously described by our team,^6^ a systematic approach was used for data extraction using a predefined excel template (Supplemental Table 2), ensuring consistency and comprehensiveness. This template captured relevant information, including title, publication year, country of origin, CKD subtype, testing method, eligibility criteria, and gene panel composition. Systematic searches were conducted in PubMed, Embase, and gray literature sources, including governmental and professional guidelines from other jurisdictions.

### Data analysis

The extracted data were categorized and analyzed based on key themes, including jurisdiction (Ontario, United Kingdom, The Netherlands, Australia, etc.), scope (eg, eligibility criteria, clinical implementation, a specific subtype of CKD, and specific population), and type of genetic testing (eg, disease-specific, comprehensive panels and/or genome-wide sequencing, which includes both exome and genome analysis). Comparative analysis highlighted trends, gaps and best practices across jurisdictions.^32^ These analyses highlighted large variations in panel content, testing eligibility criteria, and implementation strategies, providing a basis for developing evidence-based recommendations tailored to Ontario. Guidelines were evaluated for inclusion and exclusion criteria and benchmarked against international frameworks.

### Consensus process

In developing our consensus recommendations, we used a modified nominal group technique, a structured method for achieving consensus among experts. This approach involved iterative rounds of idea generation, discussion, and ranking, culminating in consensus when at least two-thirds of the panel members agreed on a recommendation. This method has been effectively used in health care research to develop complex interventions and guidelines.^33^ Draft proposals were circulated for review and discussed over a series of virtual meetings and email correspondences. Consensus was defined as agreement by at least two-thirds of the working group members.

### Generation of genetic kidney disease gene panels

The Expert Group followed an evidence-based framework (Table 2) for each disease-specific panel to achieve consensus on which genes to include on the genetic kidney disease panels. All expert group members were asked to vote for the inclusion and exclusion of genes on each disease-specific gene panel. Recommendations included comprehensive sequencing of coding regions and intron/exon boundaries for all selected genes and inclusion of copy-number variants (CNV) to ensure diagnostic accuracy.

**Table 2:** Evidence framework for gene inclusion

Resources	Evidence Threshold
Clinical Genome Resource (ClinGen)	Genes curated as Moderate, Strong, or Definitive for gene-disease validity in ClinGen
Genomics England PanelApp	Genes identified as Green using the Genomics England PanelApp and nominated by the EG member(s)
PanelApp Australia	Genes identified as Green using the PanelApp Australia and nominated by the EG member(s)
Expert Consensus	Genes for which there is supportive evidence in the literature and vetted by the EG members.

*EG*, Expert Group.

## Results

### Environmental scan and priority setting

**Figure 1:**
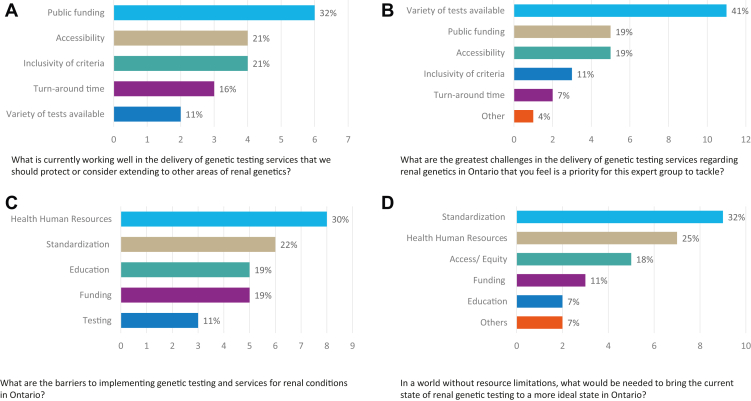
The results of the priority setting exercise administered to all members of the Renal Genetic Expert Group at an initial meeting in April 2023.

Environmental scan revealed that most of the genes known to cause CKD are not included in the gene panels available in Ontario, with <45 genes currently available in-province as either multigene testing panels or single-gene testing (Supplemental Table 3). All other gene testing requires a MOH application and pre-approval for out-of-country testing.^34^ GWS (including clinical exome and genome) sequencing is currently performed at 2 sites in Ontario through MOH funding, when patients meet specific indications for testing (Supplemental Table 4). However, few patients with CKD qualify for GWS based on the current criteria.^31^ After a priority setting exercise, limited availability of genetics tests was identified as the greatest current challenge in the delivery of kidney genetic testing in Ontario (Figure 1).

### Approach to genetic testing

**Figure 2:**
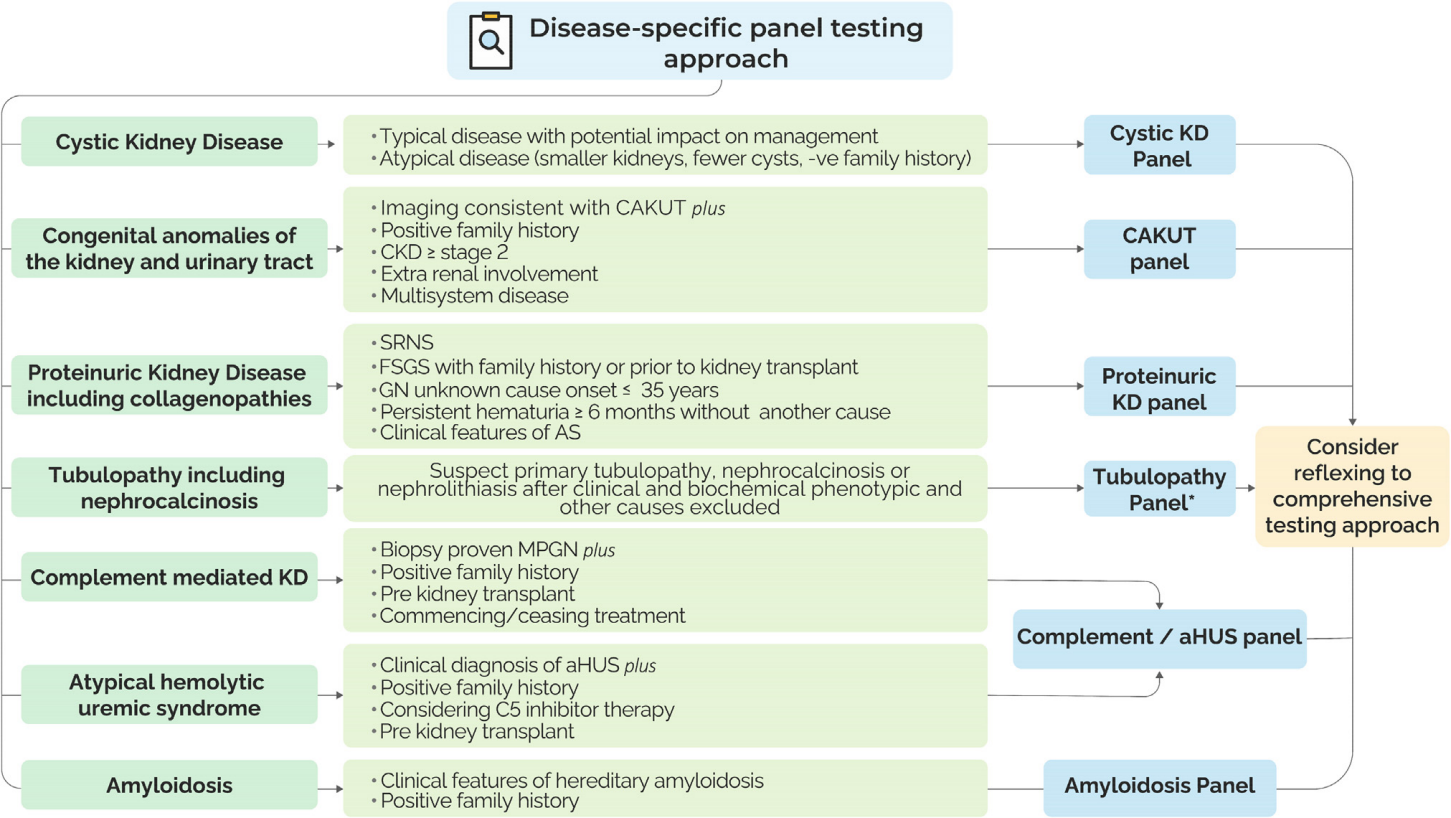
**Disease-Specific approach to genetic testing when there is a high suspicion of a certain subtype of CKD including eligibility criteria and indicated panel(s).** ∗Can consider a subpanel within the general tubulopathy panel if there is a high index of suspicion for a specific phenotype (ie, Dent Disease Sub-Panel, see Figure 5). All panel testing should include CNV analysis. *aHUS*, atypical hemolytic uremic syndrome; AS, Alport syndrome; CAKUT, congenital anomalies of the kidney and urinary tract; CKD, chronic kidney disease; CNV, copy-number variant; ESKD, end-stage kidney disease (ie, kidney failure); FSGS, focal segmental glomerulosclerosis; GN, glomerulonephritis; KD, kidney disease; MPGN, membranoproliferative glomerulonephritis; SRNS, steroid resistant nephrotic syndrome.

**Figure 3:**
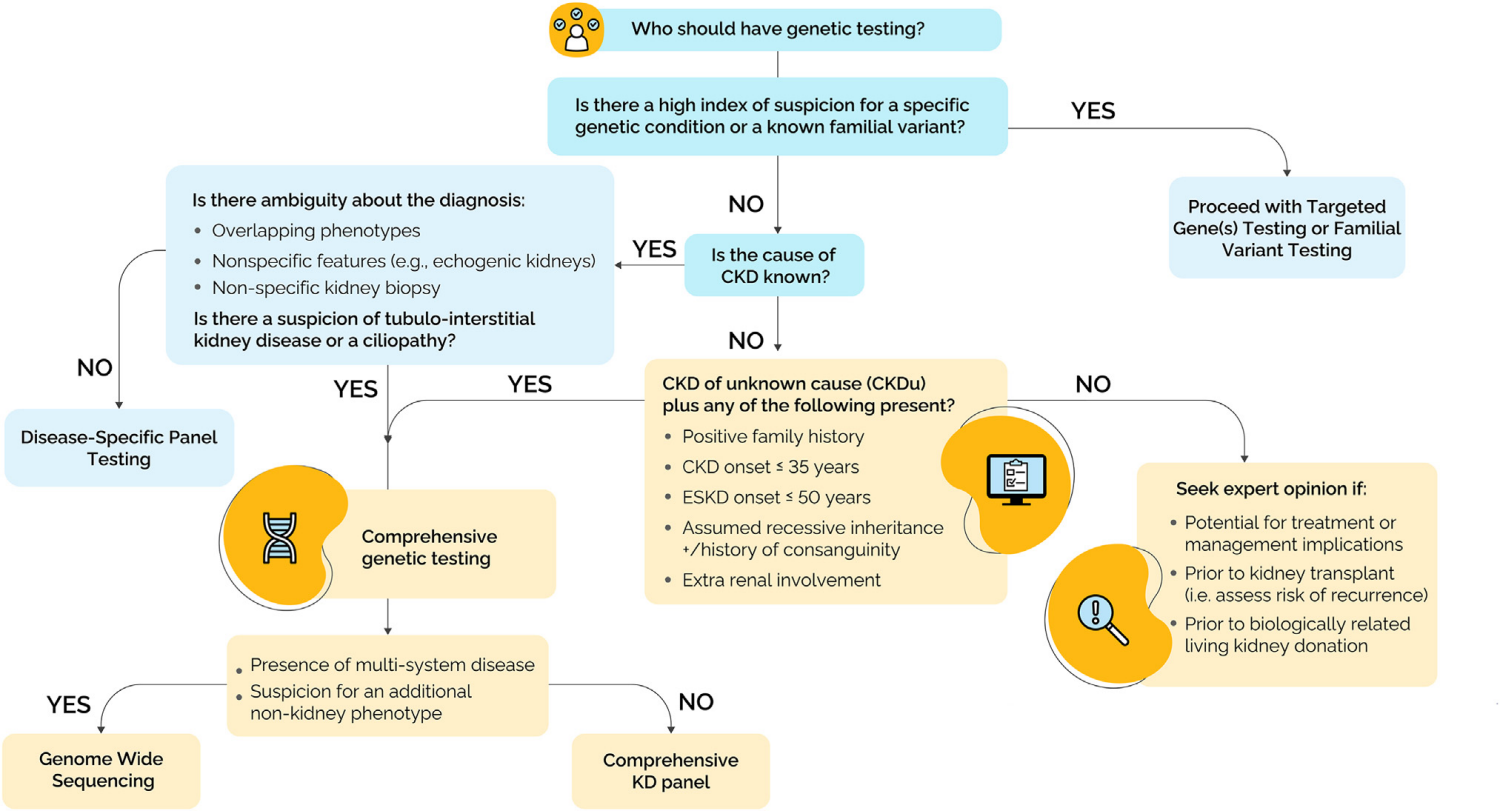
**Comprehensive approach to genetic testing.** GWS refers to both genome sequencing and exome sequencing. If childhood onset, multisystem disease, or syndromic features can consider microarray as a first-line test before GWS. All panel testing should include CNV analysis. Note that ciliopathies includes nephronophthisis. CKD, chronic kidney disease; CNV, copy-number variant; ESKD, end-stage kidney disease (ie, kidney failure); GWS, Genome-wide sequencing.

We recommend 2 major approaches to genetic testing in patients with suspected genetic kidney disease: (1) a “Disease-Specific Approach to Testing” (Figure 2) or (2) a “Comprehensive Approach to Testing” (Figure 3). When choosing the most appropriate approach for a patient and/or family, the approach should encompass all genes associated with the suspected CKD diagnosis and be sufficiently comprehensive to include all implicated kidney disease genes with important clinical phenotype overlap. The choice of panels should be driven by clinical judgement, informed by the patient’s phenotype, family history, and need for diagnostic certainty.

### Tiered genetic testing strategy

**Figure 4:**
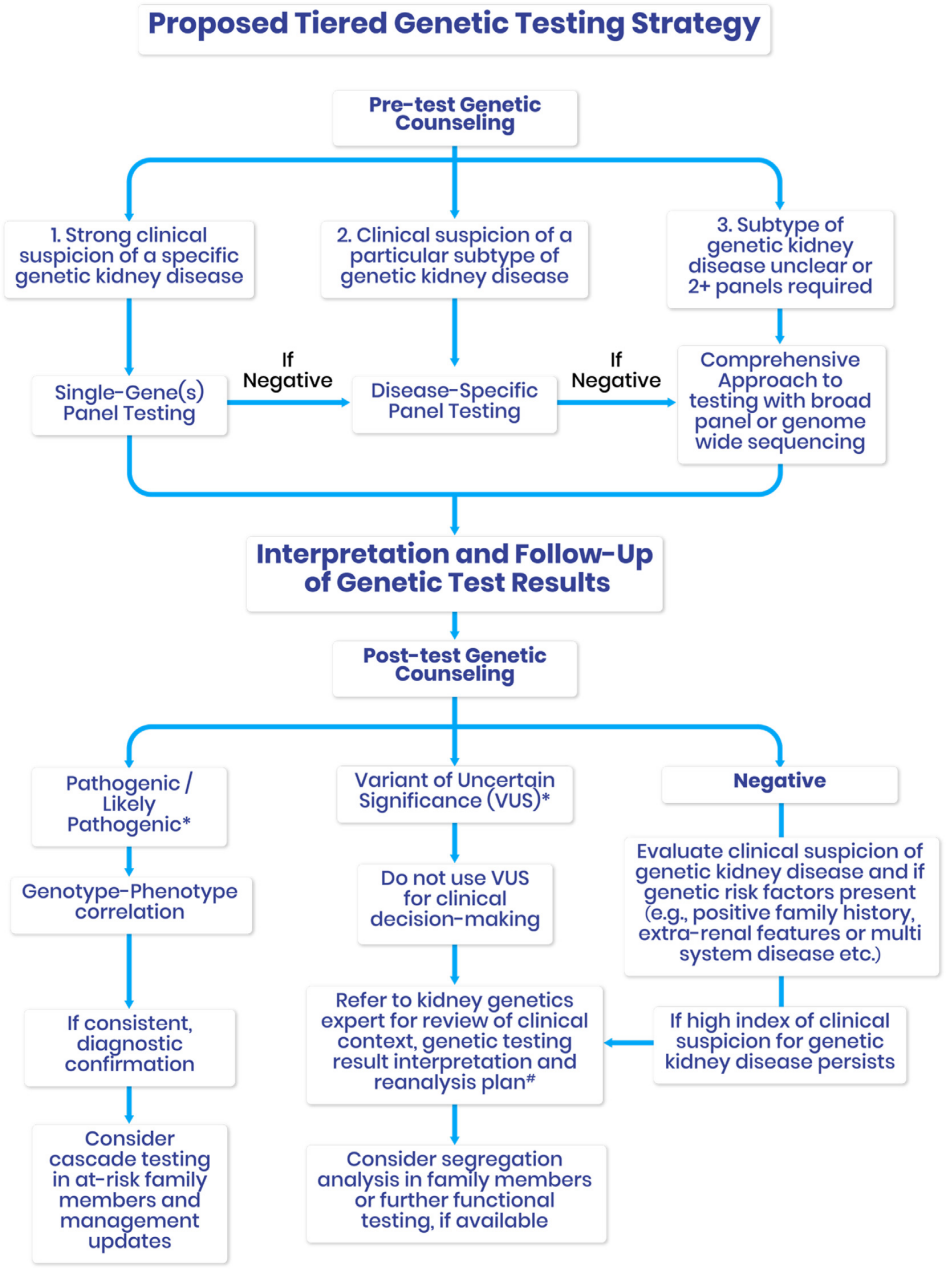
**Framework for tiered genetics testing strategy and for the interpretation and follow-up of genetic test results.** ∗As per the American College of Medical Genetics and Genomics (ACMG) Guidelines. #Reanalysis could occur at a 18 to 36 months interval guided by the clinical context.

In cases in which there is a strong clinical suspicion for a specific monogenic kidney disease, a tiered genetic testing strategy may be appropriate (Figure 4). This involves initial single-gene testing with reflex to a broader phenotype-driven or comprehensive kidney gene panel if no pathogenic variant is identified. Such an approach may optimize turnaround time and resource use, especially when the suspected gene is included in an established panel. Although this reflexive testing model is not yet widely implemented in Ontario, it is under development as part of future enhancements to provincial laboratory services.

### Suspicion of specific gene(s)

Single-gene testing is recommended for individuals when there is a high clinical suspicion for a specific genetic condition. In such cases, the clinician may directly order testing for the relevant gene(s). However, if the suspected genetic condition is unclear, a disease-specific panel or a comprehensive genetic testing approach may be more appropriate.^35^

#### Examples of targeted gene(s) testing approach

Alagille Syndrome panel^36^ (*JAG1, NOTCH2*)

Cystinosis panel^37^ *(CTNS)*

Cystinuria panel^38^ (*SLC3A1*, *SLC7A9*)

Fabry Disease panel^39^ (*GLA*)

Glomerulopathy with fibronectin deposits based on kidney biopsy report^40^ (*FN1)*

Primary Hyperoxaluria panel^35^ (*AGXT*, *GRHPR*, *HOGA1*)

Renal Cyst and Diabetes Syndrome^41^ *(HNF1B)*

#### Disease-specific approach to testing

**Figure 5:**
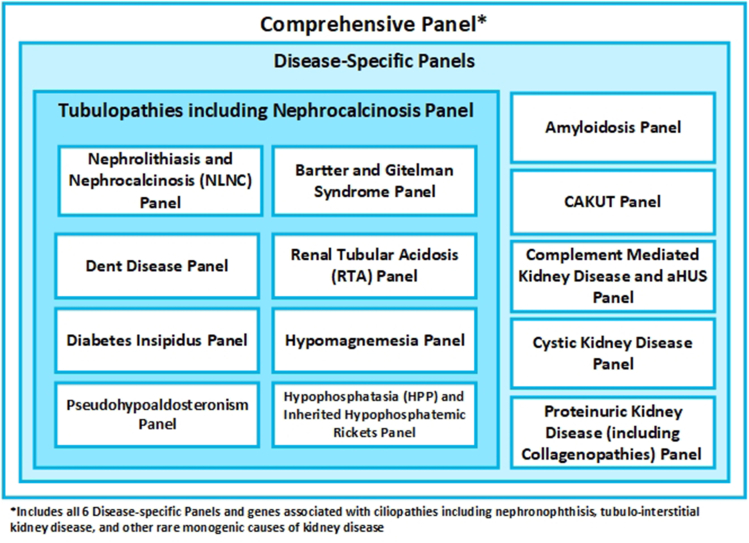
The design of the Kidney Genetic Gene Panels.

If there is a clinical suspicion regarding the subtype of CKD, a disease-specific panel testing approach can be considered (Figure 5). A description of each panel is provided below with the associated genes outlined in Table 3.

**Table 3:** Gene contents for kidney disease genetic testing panels

Panel Type Ordered (Number of Genes Included)	Genes Included on Each Panel
**Comprehensive Approach**	
**Comprehensive Kidney Disease Panel** **(346 genes including 63 additional genes not included in disease-specific panels)**	All genes currently included in the individual disease-specific panels, plus additional genes associated with ciliopathies/ nephronophthisis, genes associated with tubulo-interstitial kidney disease, and other rare monogenic causes of kidney disease as outlined below: *AHI1, ANKS6, APOA4, ARL13B, ARL6/BBS3, ARMC9, BBS1, BBS10, BBS12, BBS2, BBS4, BBS5, BBS7, BBS9, C5orf42/CPLANE1, CC2D2A, CEP104, CEP164, CEP290, CEP41, CEP83, CSPP1, CYS1, DCDC2, DYNC2H1, FAN1, IFT122, IFT172, IFT27, IFT43, INPP5E/PMPCA, INVS, IQCB1, KIAA0586, KIF7, LZTFL1, MAPKBP1, MKKS, MKS1, MUC1, NPHP1, NPHP3, NPHP4, OSGEP, RPGRIP1L, SDCCAG8, TCTN1, TCTN2, TCTN3, TMEM107, TMEM138, TMEM216, TMEM231, TMEM237, TMEM67, TRAF3IP1, TTC21B, TTC8, TULP3, WDPCP, WDR19, WDR60/DYNC2I1, XPNPEP3*
**Disease Specific Approach**	
**Amyloidosis Panel (8)**	*APOA1, APOA2, APOC2, FGA, GSN, LYZ, TTR, NLRP3*
**CAKUT Panel (98)**	*ACE, ACTG2, AFF3, AGT, AGTR1, ALMS1, ANOS1, B9D2, BMP4, BNC2, CD151, CENPF, CEP55, CHD7, CHRM3, CHRNA3, COL4A1, CTU2, CUL3, DDX59, DHCR7, DLG5, DSTYK, EXOC3L2, EYA1, FAM58A/CCNQ, FAT1, FLCN, FOXP1, FRAS1, FREM1, FREM2, GATA3, GDF6, GFRA1, GLI3, GNAS, GPC3, GREB1L, GRIP1, HAAO, HNF1B, HOXA13, HPSE2, HS2ST1, HSPA9, HYLS1, ICK/CILK1, ITGA8, JAG1, KDM6A, KIAA0753, KIF14, KMT2D, KYNU, LIFR, LMX1B, LRIG2, LRP4, MYOCD, NADSYN1, NEK8, NFIA, NIPBL, NOTCH2, NR3C2, NRIP1, OFD1, PAX2, PBX1, PLVAP, REN, RET, RMND1, ROBO1, ROBO2, ROR2, SALL1, SALL4, SCLT1, SCNN1A, SCNN1G, SIX5, STRA6, STRADA, TBC1D1, TBX18, TFAP2A, TMEM260, TRAP1/HSP90B2P, TXNDC15, WBP11, WDR35, WDR72, WNT5A, WT1, ZIC3, ZMYM2*
**Complement Medicated Kidney Disease and Atypical Hemolytic Uremic Syndrome (aHUS) Panel (12)**	*C3, CD46, CFB, CFH, CFHR1, CFHR2, CFHR3, CFHR5, CFI, DGKE, MMACHC* *C5* gene testing is not indicated for diagnosis but in treatment considerations testing can be considered as it may be associated with treatment resistance^42^. Genomic rearrangement can occur in the *CFH* and *CFHR1-5* in patients with aHUS^43^. If complex rearrangements are suspected, additional assays are required including Multiplex Ligation-dependent Probe Amplification (MLPA).
**Cystic Kidney Disease Panel (18)**	*ALG5, ALG8, ALG9, CEP55, DNAJB11, DZIP1L, GANAB, HNF1B, IFT140, KIAA0753, NEK8, OFD1, PKD1, PKD2, PKHD1, PRKCSH, SEC63, UMOD*
**Proteinuric Kidney Disease including collagenopathies (72)**	*ACTN4, AMN, APOA1, APOA2, APOC2, APOE, APOL1, ARHGDIA, CD151, CD2AP, CLCN5, COL4A1, COL4A3, COL4A4, COL4A5, COQ2, COQ6, COQ8B, CRB2, CTNS, CUBN, DAAM2, DGKE, DLC1, FAH, FAT1, FGA, FN1, GLA, GON7, GSN, INF2, ITGA3, ITSN1, ITSN2, KANK2, LAGE3, LAMA5, LAMB2/LAMC1, LCAT, LMX1B, MAFB, MAGI2, MTX2, MYH9, MYO1E, NOS1AP, NPHS1, NPHS2, NUP107, NUP133, NUP85, NUP93, OCRL, PAX2, PDSS2, PLCE1, PMM2, PODXL, PTPRO, SCARB2, SGPL1, SMARCAL1, TBC1D8B, TNS2, TP53RK, TPRKB, TRIM8, TRPC6, WDR73, WT1, YRDC*
**Tubulopathy including Nephrocalcinosis Gene Panel (100)**	*AGXT, ALPL, AP2S1, APRT, AQP2, ATP1A1, ATP6V0A4, ATP6V1B1, AVP, AVPR2, BCS1L, BSND, CA2, CACNA1D, CACNA1H, CACNA1S, CASR, CDC73, CLCN2, CLCN5, CLCNKB, CLDN10, CLDN16, CLDN19, CNNM2, CTNS, CUL3, CYP11B1, CYP11B2, CYP17A1, CYP21A2, CYP24A1, CYP27B1, CYP2R1, DMP1, ENPP1, FAM20A, FGF23, FOXI1, FXYD2, GALNT3, GATM, GCM2, GNA11, GRHPR, HNF1B, HNF4A, HOGA1, HPRT1, HSD11B2, HSD3B2, IFT74, KCNA1, KCNJ1, KCNJ10, KCNJ16, KCNJ5, KLHL3, LRP2, MAGED2, MOCOS, MT-TF, NEK1, NR3C1, NR3C2, OCRL, PHEX, PTH1R, RRAGD, RRM2B, SARS2, SCN4A, SCNN1A, SCNN1B, SCNN1G, SEC61A1, SLC12A1, SLC12A3, SLC22A12, SLC2A2, SLC2A9, SLC34A1, SLC34A3, SLC36A2, SLC3A1, SLC4A1, SLC4A4, SLC5A2, SLC6A19, SLC6A20, SLC7A7, SLC7A9, STX16, TRPM6, VDR, VIPAS39, VPS33B, WNK1, WNK4, XDH*
**Tubulopathy Sub-Panels**	
Dent Disease Panel (2)	*CLCN5, OCRL*
Diabetes Insipidus Panel (3)	*AQP2, AVP, AVPR2*
Pseudohypoaldoseronism Panel (10)	*CUL3, HSD11B2, KCNJ5, KLHL3, NR3C2, SCNN1A, SCNN1B, SCNN1G, WNK1, WNK4*
Bartter and Gitelman Syndrome Panel (11)	*BSND, CASR, CLCNKB, CLDN16, CLDN19, GNA11, HSD11B2, KCNJ1, MAGED2, SLC12A1, SLC12A3*
Renal Tubular Acidosis Panel (5)	*ATP6V0A4, ATP6V1B1, CA2, SLC4A1, SLC4A4*
Hypomagnesemia Panel (13)	*ATP1A1, BSND, CASR, CLCNKB, CLDN16, CLDN19, CNNM2, FXYD2, HNF1B, KCNJ10,SARS2, SLC12A3, TRPM6*
Hypophosphatasia Panel (11)	*ALPL, CYP27B1, CYP2R1, DMP1, ENPP1, FGF23, FAM20C, PHEX, SLC34A1, SLC34A3, VDR*

Note: Several genes are listed on more than one panel or subpanel. No disease specific panels for ciliopathies/ nephronophthisis or autosomal dominant tubulo-interstitial kidney disease are provided as the genes implicated in these subtypes of kidney disease are included in the comprehensive kidney disease panel.

### Cystic kidney disease gene panel

A cystic kidney disease panel can be considered when there is presence of 2 or more cysts in each kidney on ultrasound.^44^ Autosomal dominant polycystic kidney disease can be subdivided into classical and atypical presentations. In classical autosomal dominant polycystic kidney disease, genetic testing may be helpful for diagnostic certainty, especially at younger ages, variable disease among family members, and/or for reproductive or living kidney donor planning.^45^ Atypical cystic kidney disease is characterized by, but not limited to milder than expected cystic burden, asymmetrical cyst distribution, smaller-than-expected kidney size, or a negative family history.^46^ Genetic testing is indicated in all cases of atypical disease to confirm the diagnosis.

### Proteinuric kidney disease (including collagenopathies) panel

This panel is indicated when specific criteria are met. These include steroid-resistant nephrotic syndrome at any age^47^^,^^48^ and proteinuria with focal segmental glomerulosclerosis or diffuse mesangial sclerosis on kidney biopsy, particularly in cases with a positive family history of CKD, an unclear cause of proteinuria, or when a kidney transplant is planned and genetic testing is needed to assess posttransplant risk of recurrence.^49^, ^50^, ^51^, ^8^ Additionally, this panel is recommended for glomerulonephritis of uncertain etiology, when there is clinical suspicion of a genetic cause.^23^^,^^24^ It is also indicated in cases of persistent hematuria lasting more than 6 months with no obvious cause, especially when accompanied by a positive family history of hematuria or biopsy features suggestive of Alport Syndrome or Alport Disease. Furthermore, patients with clinical features suggestive of Alport syndrome, such as hearing impairment and/or eye pathology, may also benefit from this genetic evaluation.^42^^,^^50^^,^^51^

### Congenital anomalies of the kidneys and urinary tracts panel

Congenital anomalies of the kidneys and urinary tracts (CAKUT) is defined as any abnormalities in the structure, function, size, shape, or position of the kidney and/or genitourinary tract.^43^ Genetic testing is indicated in patients with CAKUT and at least 1 of the following: positive family history of CAKUT or unexplained CKD in a biologically related family member,^43^ impaired kidney function defined as a CKD Epidemiology Collaboration stage CKD ≥2 or higher,^52^ extrarenal features of disease, and/or evidence of multisystem disease.^53^ For childhood-onset disease with multisystem involvement, CNV analysis using microarray may be considered before CAKUT panel testing, given the high rate of structural variants associated with disease.^54^^,^^55^ If there is evidence of multisystem disease, the ordering clinician can consider a comprehensive genome-wide sequencing approach as the first-line test (see below).

### Amyloidosis gene panel

An amyloidosis panel is indicated when there is clinical suspicion for the diagnosis due to the presence of abnormal amyloid deposits on a pathological specimen (ie, kidney or skin biopsy) and clinical features suggestive of hereditary amyloidosis,^56^ when other secondary causes of amyloidosis have been ruled out.^29^ Clinical features suggestive of amyloidosis include but are not limited to restrictive cardiomyopathy,^57^ autonomic and peripheral neuropathy,^58^ gastrointestinal involvement,^59^ and positive family history of amyloidosis.^60^

### Complement-mediated kidney disease and atypical hemolytic uremic syndrome panel

A complement-mediated kidney disease and atypical hemolytic uremic syndrome (aHUS) panel is recommended for patients with CKD caused by dysregulation of the complement system.^61^ Such patients may present with biopsy-confirmed membranoproliferative glomerulonephritis, a glomerular injury pattern characterized by hypercellularity and thickening of the glomerular basement membrane.^62^ The panel is also indicated in cases of complement-mediated glomerulonephritis with either a positive family history of the condition and/or if there is a need for genetic diagnosis to guide transplant management or treatment planning.^61^ aHUS is characterized by acute kidney injury, thrombocytopenia, microangiopathic hemolytic anemia, and a negative Coombs test.^63^ Genetic testing is recommended in aHUS patients, when there is a positive family history of aHUS suggestive of a hereditary component. Of note, familial forms account for approximately 20% of cases, defined by at least 2 affected family members.^64^ Additionally, genetic testing can guide C5 inhibitor therapy because identifying pathogenic variants in complement genes informs the use of eculizumab or ravulizumab, which effectively inhibit terminal complement activation.^64^, ^65^, ^66^ Genetic insights also play a critical role in kidney transplant planning because certain genetic variants are linked to a higher risk of posttransplant recurrence, aiding in risk assessment and perioperative management.^67^

### Tubulopathies, including nephrocalcinosis panel

Renal tubulopathies are defined as diseases of the renal tubule which lead to dysfunction in either water, electrolytes, and/or acid-base homeostasis.^68^ Genetic testing should be considered in cases in which there is a clinical suspicion of a specific tubulopathy, after full clinical and biochemical phenotyping has been performed, and exclusion of secondary causes.^69^ A dedicated disease-specific panel should be considered when there is a high index of clinical suspicion for a specific tubulopathy (eg, Dent Disease, Renal Tubular Acidosis, Bartter syndrome, Gitelman syndrome, hypomagnesemia, pseudohypoaldosteronism, and/or diabetes insipidus (Figure 5). For other rare tubulopathies, expert consultation is recommended. In patients with radiologically confirmed nephrocalcinosis, genetic testing can be performed after excluding acquired causes.^70^ Genetic testing is indicated in recurrent nephrolithiasis when a genetic etiology is suspected,^71^ particularly with a positive family history or pediatric onset.^72^

### Tubulo-interstitial kidney disease and nephronophthisis

Tubulo-interstitial kidney disease is a genetically heterogeneous disorder characterized by progressive tubulo-interstitial damage, subtle clinical features (progressive CKD, bland urine sediment), and a delayed onset of symptoms in adulthood, with an autosomal dominant pattern of inheritance.^73^ Given its nonspecific clinical presentation, we recommend a comprehensive approach to testing that includes, but is not limited to, the following genes: *UMOD*, *MUC1*, *REN*, *HNF1B*, *DNAJB11*, *SEC61A1*, and *APOA4* (Figure 3).

Nephronophthisis is a genetically distinct yet clinically overlapping tubulointerstitial kidney that typically presents in childhood or adolescence and follows a predominately autosomal recessive inheritance pattern. It leads to progressive renal fibrosis and cystic changes at the corticomedullary junction and may be associated with extrarenal features or multisystem disease.^74^ Given the nonspecific clinical presentation of this condition, we recommend a comprehensive approach to genetic testing (Figure 3). Genetic testing should be considered for patients with CKD when there is clinical suspicion of a genetic etiology based on early onset, positive family history, syndromic features, and/or unexplained progression of disease.

### Comprehensive approach to testing

When the cause of CKD is unknown or unclear, a comprehensive testing approach should be considered,^10^^,^^23^^,^^24^ ideally performed on an exome or genome sequencing backbone. The initial comprehensive panel should include all genes from the relevant subtypes of CKD included in the Disease-Specific Approach Panels (Figure 5). Reflexing refers to the process in which initial disease-specific panel testing is performed on a GWS platform, with the option to bioinformatically extend the analysis to a more comprehensive panel encompassing more kidney disease genes or, indeed, all known disease causing genes.^75^ This broader analysis should be considered when the initial results are negative or inconclusive and the likelihood of a genetic etiology is considered high. CNV analysis should always be included, whether using a gene panel or a GWS approach. In some cases, CNV analysis using microarray may be considered, particularly when there is clinical suspicion of a specific diseases where deletions or duplications are known to cause disease (eg, *NPHP1*, *HNF1B*).

### Criteria for comprehensive approach to testing

#### Chronic kidney disease of unknown cause

Chronic kidney disease of unknown cause **(**CKDu) is defined as an estimated glomerular filtration rate (eGFR) of less than 60 mL/min/1.73 m^2^ and the presence of kidney damage (including hematuria, proteinuria, or structural anomalies of the kidney and/or genitourinary tract), lasting for 3 months or longer, for which no definitive primary kidney disease is identified.^23^^,^^24^ This includes individuals with both positive and negative family histories of kidney disease.^7^ The diagnostic yield from genetic testing for CKDu ranges from 12% to 56%.^12^^,^^76^, ^77^, ^78^, ^79^, ^80^ Currently, genetic testing is recommended for individuals diagnosed with CKDu who are ≤35 years old and have stage 3 or higher CKD or kidney failure onset before the age of 50 years.^29^ Increasingly, data demonstrate the utility of genetic testing for individuals outside of these age ranges.^9^^,^^10^ Therefore, in line with KDIGO recommendations, that there should be no upper age limit for monogenic kidney disease,^12^ we suggest that genetic testing can still be considered following expert consultation and/or if additional clinical information is available that may impact management. Any individual with CKDu diagnosed at ≤18 years, without an identifiable cause, should be considered for genetic testing.^81^

#### CKD with overlapping phenotypes

If there is ambiguity regarding the underlying subtype of CKD,^53^ if a phenocopy is suspected and more than one disease-specific panel could apply,^10^ a custom panel can be considered. If multiple disease-specific panels (≥2) are anticipated from the outset, a comprehensive approach to testing should be considered as the initial test, with data suggesting that this may be the more efficient and cost-effective approach to testing.^16^^,^^19^^,^^20^ For example, comprehensive testing should be considered as the initial testing approach in patients with persistent unexplained hyperechogenicity (eg, loss of corticomedullary differentiation),^82^ which may be suggestive of a number of different subtypes of CKD including nephronophthisis, ciliopathies or tubulo-interstitial kidney disease. Equally, if a kidney biopsy is not feasible because of small size of the kidneys or advanced stage disease and/or if a kidney biopsy report is inconclusive or shows a nonspecific pattern of injury,^25^ comprehensive testing should be considered.

#### Positive family history

Genetic testing should be considered for patients with CKD who have a first- or second-degree relative with CKD.^6^ Genetic assessments should still be considered in cases in which the clinical presentation is highly suggestive of a genetic kidney disease but family history is unknown or negative (eg, the patient was adopted).^7^

#### History of consanguinity

Genetic testing should be considered in individuals with suspected genetic kidney disease when there is a known or suspected history of consanguinity between the parents of the affected patient. The inheritance pattern in the family will typically be consistent with autosomal recessive inheritance; however, families with multiple loops of consanguinity could demonstrate pseudo-dominant inheritance.^83^

#### Extrarenal involvement

In individuals with CKD who also exhibit features of extrarenal involvement or multisystem disease, genetic testing should be considered.^7^^,^^84^

### Criteria for GWS

GWS encompasses both exome and genome sequencing and can be considered in the following circumstances as the initial genetic testing approach:

• Dual diagnosis suspected: if there is suspicion of a non-kidney-related genetic disease, in addition to CKD, and the conditions in question would not be covered by a comprehensive kidney disease gene panel.
• Presence of multisystem disease: in cases in which CKD is accompanied by multisystem involvement.
• Broad genetic differential diagnosis : if ≥2 disease-specific gene panels are indicated, GWS may be the more cost-effective and efficient approach to testing.^16^^,^^20^

### Special considerations

#### *APOL1* testing in symptomatic individuals

In alignment with the American National Kidney Foundation Working Group, *APOL1* testing should be included as part of a multigene panel offered to all patients, regardless of race or ethnicity. This approach acknowledges the inaccuracies of self-reported race and the limitations of using social constructs, such as race and ethnicity, to infer biological classifications such as genetic ancestry.^85^^,^^86^ Given the challenges in identifying or quantifying genetic ancestry, *APOL1* testing should not be restricted based on reported or perceived race or ethnicity because these categories are inconsistent proxies for genetic risk.^87^^,^^88^ Accordingly, we recommend incorporating *APOL1* testing in the Proteinuric and Comprehensive Kidney Disease Panels to ensure an inclusive and equitable approach to genetic risk assessment. The recommendation aligns with the National Kidney Foundation and the American Society of Nephrology.^85^ By adopting a universal, race-neutral framework, the Ontario models aid to address longstanding concerns about the inequities of race-based testing in CKD diagnosis and treatment.

#### *MUC1* (*Mucin-1*) testing for autosomal dominant tubulo-interstitial kidney disease

*MUC1* testing is not currently available on any commercially available sequencing platform, although data are emerging on enhanced detection strategies.^89^ At present, commercially available sequencing platforms do not detect the specific cytosine insertion in the variable number of tandem repeat region of the *MUC1* gene.^90^ If autosomal dominant tubulo-interstitial kidney disease (ADTKD) due to a *MUC1* variant is suspected, please seek kidney genetic expert advice before proceeding with testing.^91^ ADTKD-*MUC1* kidney disease should be considered in individuals with adult-onset CKD, with a bland urine sediment (ie, negative urine dipstick for blood and protein) and a positive family history of CKD in an autosomal dominant pattern of inheritance.^92^

#### Individuals in whom a genetic diagnosis is needed

Certain populations may require a genetic confirmation of CKD to guide treatment planning, inform management decisions, and to provide accurate family and reproductive counselling.^93^^,^^94^ Below are examples of situations in which genetic testing should be considered, along with general guidelines for when testing is indicated.

#### Reproductive planning

For individuals with CKD or a family history of kidney disease who are planning for reproduction, genetic testing may be offered to assess potential genetic risks in offspring.^95^ Expert consultation is recommended to help guide decision making, ensuring appropriate counselling and informed reproductive choices.

#### Treatment implications

Genetic testing may have important treatment implications.^9^ For example, identifying a genetic cause of CKD can inform treatment strategies, potential therapeutic interventions, and kidney transplant planning.^6^^,^^9^ Specific conditions, such as Focal Segmental Glomerulosclerosis or Alport syndrome, may influence how patients are managed both before and after transplant.^50^ Genetic testing should be considered in any individual with CKD in whom confirmation of a genetic diagnosis may have treatment implications.

#### Rapid genetic testing or need for rapid diagnosis

Genetic testing should be considered when a rapid diagnosis is needed, particularly for

##### Acutely Unwell Individuals

In children or adults whom monogenic kidney disease is suspected as the primary cause of disease, rapid genetic testing can be considered to confirm the diagnosis and guide urgent clinical decisions.^96^, ^97^, ^98^ For example, in patients with clinical signs of primary hyperoxaluria type 1 post kidney transplant, the genetic diagnosis should be confirmed so that lumasiran can be administered to prevent further oxalate buildup, which can damage the transplanted kidney and other organs.^99^, ^100^, ^101^

##### Rapid Changes in Management

If genetic testing will lead to an immediate change in treatment,^102^ such as informing decisions about kidney transplant, therapeutic intervention,^103^ or prenatal testing for an at-risk pregnancy,^104^ testing should be performed as part of the clinical decision-making process

### Neonatal screening with multicongenital anomalies

GWS testing should be considered in neonates presenting with multiple congenital anomalies, including kidney anomalies consistent with the diagnosis of CAKUT.^105^ Early genetic testing can help identify the underlying genetic conditions that may guide treatment and management.^106^

### Kidney transplantation

For individuals undergoing kidney transplantation, genetic testing should be considered in the following scenarios:

• End-stage kidney disease onset before age 50 especially when the cause of CKD is unknown.^107^
• Suspected genetic condition: if a heritable kidney disease is suspected, genetic testing should be considered to inform transplant planning.^77^
• Clinical implications after transplant: pretransplant genetic testing in individuals in whom a confirmed genetic diagnosis may inform the risk of disease recurrence posttransplant.^77^

Genetic testing should be considered in the affected individual (the person with CKD who requires a kidney transplant) to confirm specific variants, with specific variant confirmation in at-risk family members. This can help guide the selection of potential biologically related living kidney donors.^108^ Comprehensive, nontargeted testing in unaffected family members wishing to proceed with living kidney donation, is not recommended at this time.^109^

### Implementation considerations

Although this work focuses on establishing standardized eligibility criteria and curated panel content, structured implementation planning is essential to ensure real-world uptake and impact. Key next steps will include piloting these recommendations in select clinical settings, integrating knowledge translation strategies to support clinician engagement, and evaluating outcomes such as test uptake, diagnostic yield, and impact on care pathways. These efforts will require close collaboration between the PGP, nephrology and genetics clinics, and implementation science experts and are currently under development. Below, we have outlined some of the initial implementation considerations.

#### Service delivery

Genetic testing has historically been provided after genetic counselling by qualified geneticists, genetic counsellors, or physicians with specialized training and expertise in genetics or those diagnosing and treating genetic kidney disease. To improve timely and equitable access for patients with CKD, the standard is shifting toward mainstreaming genetic testing—allowing nongenetics physicians involved in the diagnosis and management of kidney disease to order genetic tests.^110^ This approach facilitates earlier diagnosis and personalized treatment but requires that ordering physicians have a clear understanding of gene-based management guidelines to ensure appropriate pre-test counselling, test selection, result interpretation, and follow-up care. Ideally, these clinical pathways should be developed in collaboration with local kidney genetics experts to optimize patient outcomes and integrate genetic insights into routine nephrology practice.^10^^,^^13^^,^^14^^,^^79^

#### Prior genetic testing

Before initiating genetic testing related to kidney disease, the ordering clinician must confirm whether genetic testing has been performed previously on the index patient or any biologically related family members. If prior testing has been conducted, every effort should be made to obtain and review the results before proceeding with further testing to avoid redundant testing and to ensure comprehensive interpretation. If prior testing has been performed using a GWS backbone (ie, exome or genome sequencing), additional panel testing is generally unnecessary. In such cases, the clinician should consider reanalysis using a virtual, bioinformatic panel approach or, if clinically indicated, a reanalysis of the GWS data.^75^^,^^111^ Evidence suggests reanalysis at 18 to 36 month intervals, especially if new clinical information is now available that could inform or refine the interpretation.^112^, ^113^, ^114^, ^115^

#### Pre- and posttesting genetic counselling

All genetic testing should be accompanied by pre- and posttest counselling, particularly when broad testing strategies are used that increase the likelihood of detecting variants of uncertain significance (VUS). Pretest counselling prepares patients for uncertain or inconclusive results, ensures informed consent and outlines potential test limitations. After the disclosure of genetic test results, patient with positive genetic testing results should be offered counselling to discuss implications for themselves and at-risk relatives.^116^ Posttest counselling is essential for interpretating results and planning follow-up, particularly when no diagnosis is reached or when VUS findings are reported. A proposed framework for managing negative or inconclusive results, including reanalysis, recontact and expanded testing is shown in Figure 4. Although formal counselling is currently provided by genetics professionals, nephrologists are increasingly initiating testing and communicating results.^110^ To support nongenetic practitioners, structured decision aids, consent tools, and referral pathways are under development and should be embedded into standard care.

### Cascade testing in affected individuals

When a pathogenic or likely pathogenic variant is confirmed in an individual with known CKD,^117^ cascade testing is recommended for at-risk biologically related family members. If genetic testing has already been conducted in a biologically related family member, every effort should be made to obtain the testing report to confirm the familial variant of interest.^118^ To minimize the risk of false negatives—such as those caused by allele dropout, which can occur in Sanger-based testing—it is advisable to perform testing in the same laboratory that analyzed the affected family member. Alternatively, the proband’s sample should be sent alongside the patient's sample as a positive control. Conducting testing in the same lab also helps prevent discrepancies in variant classification.

### Cascade testing in an unaffected family members (ie, predictive testing)

Asymptomatic family members often seek genetic testing because of a known family history of kidney disease or after the identification of a pathogenic or likely pathogenic variant in another family member with CKD. Predictive testing—defined as testing an asymptomatic, biologically related individual—should be conducted only after appropriate genetic counselling and by clinicians with expertise in heritable kidney diseases. Interdisciplinary pathways and new models of service delivery should be supported to ensure timely, equitable access to assessment and testing. These pathways should align with locally established protocols for risk assessment, results disclosure and long-term follow-up. The genetic test results from the affected index individual should always be reviewed before testing relatives, with the goal of performing familial variant testing. Pretest counselling is essential to address potential outcomes, including variants of uncertain significance and the broader medical, psychological, and insurance-related implications of testing. Posttesting counselling should be offered to guide interpretation and support appropriate clinical or surveillance strategies where relevant.

### Testing family members for VUS

A VUS is defined by the American College of Medical Genetics Guidelines as a genetic variant with insufficient or conflicting evidence regarding its pathogenicity and therefore does not meet criteria for classification as benign, likely benign, pathogenic, or likely pathogenic.^117^ A VUS should not be used in clinical decision making.^119^ Efforts to resolve the classification of a VUS to either “pathogenic” or “benign” are essential and may include familial segregation studies, functional assessments, and reassessment of VUS over time incorporating new data as they become available.^117^^,^^120^ Reanalysis can occur at regular intervals, typically every 18 to 36 months, to ensure the variant’s classification remains up to date.^13^ If a VUS is identified in a gene associated with a heritable kidney condition, it should be reviewed by a genetic professional or a clinician experienced in variant interpretation. In certain cases, testing affected family members for a VUS may assist in determining if the VUS segregates with the disease. It is important to note that, although cosegregation analysis can be useful, in isolation, it is often not sufficient to establish pathogenicity in the absence of other supporting data (eg, the minor allele frequency is low in gnomAD). In specific situations, testing unaffected family members may be appropriate.^119^ For example, (1) to determine if a variant is de novo (not inherited) when a VUS is suspected to contribute to disease or (2) in autosomal recessive conditions, testing can help determine if the variants are inherited in trans (on opposite alleles) or in cis (on the same allele).

### Negative or inconclusive results and expanded testing

If no genetic cause for CKD is identified through a disease-specific panel testing approach, reflex testing to a broader approach—such as a comprehensive kidney disease gene panel or GWS, including exome or genome analysis—should be considered, particularly when clinical suspicion remains high (eg, a positive family history, early-onset disease, or extrarenal features of disease) (Figure 4). Ideally, in these cases, the initial gene panel testing should be conducted on a GWS platform, which enables seamless “reflex” to a broader, virtual panel or full exome/genome analysis without requiring a new sample. This approach maximizes diagnostic yield and allows for tiered analysis based on clinical findings. When the cause of CKD is unclear from the outset, comprehensive testing maybe the most appropriate first-tier investigation (Figure 3).^75^

#### Testing access and funding in Ontario

Canada operates under a universal health care system, with access to genetic testing varying by province. In Ontario, genetic testing that meets provincial eligibility criteria is publicly funded and performed through designated provincial labs. Currently there are 8 approved gene panels covering 43 different genes available for clinical use (Supplemental Table 3). Any clinically indicated genetic test beyond these panels requires out-of-country approval from the MOH until panel repatriation is complete. Efforts are underway to expand the availability of recommended panels within Ontario, with the goals of streamlining access and improving patient care. In parallel, GWS is currently available at 2 Ontario sites, for patients who meet specific indications (Supplemental Table 4).

### Interpretation of genetic test results

Results from genetic testing should be interpreted according to the American College of Medical Genetics and Genomics variant classification system, which includes 5 categories: pathogenic, likely pathogenic, VUS, likely benign, and benign.^117^ Only variants classified as pathogenic or likely pathogenic and consistent with the patient’s phenotype should be considered diagnostic. In these cases, the result may inform clinical management, cascade testing, and patient education.

VUS should not be used to guide clinical decisions. These findings require careful contextualization and should prompt review by a genetic counsellor and an expert in genetic variant interpretation. Reinterpretation over time may lead to reclassification, and patients should be informed of the possibility of recontact if variant classification changes. Figure 4 provides a decision-support framework to help nongenetics providers navigate result interpretation and determine when to refer for specialty input.

#### CNV analysis

All genetic testing, including gene panels and GWS, should be designed to capture exon and gene level deletions and duplication CNV in all genes. If these regions are not adequately covered by the initial testing, alternative methods, such as multiplex ligation probe amplification (MLPA) or microarray analysis, for larger structural variants, may be considered.

#### Uncertainty in interpretation of genetic testing results

If there is any uncertainty in interpreting the genetic report, the ordering clinician should seek expert consultation through resources and new models of care, such as eConsult,^121^, ^122^, ^123^ variant review boards,^12^^,^^124^ or direct referral to a clinical genetics or kidney genetics clinic.

#### Equity considerations and the eGFR calculations

The PGP aims to develop equity-informed recommendations recognizing the impact of health policy on outcomes, especially in underserved populations. It limits the use of eGFR and kidney disease stages as eligibility criteria because of concerns about race-based calculations, which have delayed diagnoses and specialty care for CKD patients.^125^ Instead, eligibility for genetic testing is based on comprehensive kidney function assessment. This shift not only ensures equitable access to genetic testing but also reinforces the framework’s commitment to advancing precision medicine while promoting fairness in patient care.

## Discussion

We present a comprehensive framework for standardized genetic testing for CKD to be implemented in Ontario, Canada. Through a combination of priority-setting exercises and an environmental scan, the Renal Genetics Expert Group^27^ identified significant gaps in genetic testing availability for CKD within the province. Currently, fewer than 45 genes are included in provincial gene panels, despite several hundred genes being associated with CKD.^6^ To address this disparity, the PGP of Ontario Health convened the Renal Genetics Expert Group to develop evidence-based recommendations. These recommendations encompass genetic testing eligibility criteria, optimal testing methodologies, and the design of both disease-specific and comprehensive gene panels. The resulting framework aims to bridge gaps in testing access, promote earlier and more accurate diagnoses, improve clinical outcomes, and support the delivery of personalized health care for individuals with CKD in Ontario.

The findings of this working group have substantial implications for both clinical practice and health policy. The proposed genetic testing framework effectively addresses critical gaps in CKD care, particularly the limited access to genetic testing for patients with CKD. By establishing clear eligibility criteria, the guidelines prioritize timely access to testing for high-risk groups, such as those with early-onset CKD,^81^ a positive family history,^6^ or unexplained etiology.^23^^,^^24^ These recommendations reinforce the role of genetic testing as an integral part of the diagnostic pathway for CKD. Beyond providing a molecular diagnosis, genetic testing has far-reaching clinical implications, including informing treatment planning, guiding kidney transplant decisions, and facilitating family counselling.^9^^,^^10^^,^^77^

A unique feature of this framework is its emphasis on a dual testing strategy: the disease-specific approach and the comprehensive approach. The disease-specific approach tailors genetic testing to the patient’s clinical presentation, targeting specific genes associated with the observed phenotype. In contrast, the comprehensive approach encompasses all genes implicated in CKD, making it particularly valuable for cases in which the etiology is unclear. By combining these approaches, the framework maximizes diagnostic yield, especially for patients with ambiguous phenotypes or those requiring timely interventions.

This work also reflects emerging global trends, as seen from countries such as the United States,^9^^,^^12^^,^^126^ Australia,^13^ the United Kingdom,^29^ and across Europe,^79^ where similar initiatives are being undertaken to integrate genetic testing into the care pathway for patients with CKD. Similar to frameworks established in Australia and the Kid-GEN Group,^46^ the Ontario guidelines emphasize reflex testing. This approach allows patients who receive negative results from a disease-specific panel to be seamlessly transitioned to a more comprehensive genetic test. Reflex testing minimizes diagnostic delays, reduces the need for repeat testing, and streamlines the patient care process.^75^ Furthermore, this approach is supported by evidence from prior studies demonstrating the cost-effectiveness and clinical utility of comprehensive testing strategies.^16^^,^^75^

Another notable strength of the recommendations is their integration of equity considerations. The framework emphasizes the importance of avoiding race-based eGFR calculations, which have historically contributed to disparities in CKD care.^127^ Instead, Ontario Health advocates for the adoption of race-free eGFR equations in clinical practice,^128^ a change expected to promote equity by reducing barriers to care and ensuring fair access to genetic testing for all populations. The findings of this initiative align closely with international guidelines, such as those from KDIGO, which advocate for incorporating genetic testing into the diagnostic pathway for CKD.^12^ KDIGO emphasizes the importance of early testing, particularly for patients with an unknown etiology or a family history of kidney disease. This initiative expands on KDIGO’s guidance by providing detailed eligibility criteria and a robust testing framework specifically tailored to the unique needs and structure of Ontario’s universal health care system.^12^ By addressing local gaps while adhering to international standards, this work represents a significant step forward in advancing precision medicine for CKD.

The policy implications of this work include the establishment of a province-wide framework for genetic testing in Ontario. Through this initiative, Ontario Health and the PGP aim to achieve several key objectives:

1. Improved diagnostic efficiency through the provision of comprehensive genetic testing approaches that offer more efficient and streamlined pathway to diagnosis, especially in complex cases in which the cause of CKD is unknown.
2. Equity in care by shifting away from race-based eGFR calculations to promote fair access to genetic testing for all patients, especially those from historically underserved populations.
3. Cost-effectiveness through early detection of genetic causes of CKD to ultimately reduce the economic burden on the health care system by preventing or slowing the progression to kidney failure,^22^ thereby avoiding costly dialysis and transplantation.
4. Clinical utility that extends beyond diagnosis to genetic testing supporting treatment planning, donor selection for transplantation, and cascade testing of at-risk family members.

From a clinical perspective, the recommendations encourage nephrologists, genetic counsellors, and transplant physicians to adopt a proactive approach to genetic testing, rather than the traditional model where testing occurs late, if ever, in the diagnostic pathway for patients with CKD.^10^ This shift is supported by studies showing that genetic diagnosis can affect clinical management in over 90% of confirmed cases.^9^

Although the proposed framework is comprehensive, there are several limitations. First, access and infrastructure to support the expansion of genetic testing services are not fully addressed. As a result, the availability of these services may be restricted, particularly in rural or underserved areas of Ontario. Addressing this gap will require investments in infrastructure, telehealth support, and the development of streamlined referral pathways to ensure equitable access across the province. Second, variant interpretation poses ongoing challenges, particularly for nongenetic clinicians. These variants often necessitate additional specialist analysis to classify them as pathogenic or benign.^129^ This ambiguity can complicate clinical decision making and may require family testing or periodic reanalysis of test results as new evidence becomes available.

The education and training of nongenetic clinicians, such as nephrologists, is a major priority that is paralleled in other internal medicine specialties (eg, oncology, cardiology, and hematology).^130^, ^131^, ^132^ Ensuring that nongenetic clinicians can perform pre- and posttest counselling for patients and interpret genetic testing results will necessitate the development of targeted educational resources and training programs. This initiative will also require additional infrastructure to support education and outreach efforts. Finally, although early genetic testing has demonstrated cost-effectiveness,^16^ the upfront costs of GWS may present financial challenges for the health care system. Sustained funding and strategic investments will be essential to ensure these testing programs’ scalability, spread, and long-term sustainability.

With these limitations in mind, we propose several future directions for research and policy development. Longitudinal studies are needed to assess the long-term impact of early genetic testing on patient outcomes, particularly in delaying progression to kidney failure. Although cost-effectiveness has been demonstrated,^19^ further analysis is required to refine the cost-benefit ratio of comprehensive gene panels and GWS approaches. Future efforts should focus on incorporating genetic testing results into electronic health records to enhance integration into both the diagnostic and care pathways for CKD patients. This integration will facilitate clinical decision making and improve coordination between nephrologists, geneticists, and kidney transplant teams. Additionally, given the broader implications of genetic testing beyond individual patients, further work is needed to increase awareness of its role in family planning and cascade testing, emphasizing its importance for identifying at-risk family members and guiding reproductive decisions.

### Conclusion

In summary, this document provides recommendations related to the eligibility criteria for genetic testing, practical approaches to clinical implementation, and evidence-based science for the affected patient with CKD. This work establishes a robust framework for the standardization of genetic testing in CKD in Ontario, providing a template that other jurisdictions can adopt to facilitate implementation. These guidelines aim to bridge diagnostic gaps and deliver personalized care to CKD patients by defining clear eligibility criteria, testing frameworks, and equity-focused approaches. The Ontario framework aligns with international best practices, fosters health equity, and promotes cost-effective care. Future priorities should include the operationalization of these recommendations, expansion of testing infrastructure, and comprehensive training programs for nongenetics clinicians to ensure widespread adoption. This initiative highlights the transformative potential of early and equitable genetic testing in CKD management, offering a pathway to improved diagnostics, better clinical outcomes, and enhanced patient-centered care. The framework not only addresses the needs of patients in Ontario but also serves as a scalable model for other regions, underscoring the pivotal role of genetic testing in modern nephrology.

## Data Availability

Additional data will be available upon request.

## Declaration of AI and AI-Assisted Technologies in the Writing Process

AI was only used in spell check, grammar, spelling, and references.

## Conflict of Interest

The authors declare no conflicts of interest.
