# Supplementary material for: Framework for standardized genetic testing recommendations for chronic kidney disease in Ontario
Source: Genet Med Open. 2025 Jul 5;3:103442. doi: 10.1016/j.gimo.2025.103442 (PMC12446555; doi:10.1016/j.gimo.2025.103442)
Supplement: Supplemental Data [file mmc1.pdf]

## Supplementary Section

Table S1. Database search strategy.

| Database | Concept | Terms |
|---|---|---|
| PubMed | Chronic kidney disease | (chronic kidney[tw] OR chronic renal[tw] OR chronic glomerul*[tw] OR chronic nephro*[tw] OR progressive kidney[tw] OR progressive glomerul*[tw] OR progressive nephro*[tw] OR diabetic kidney[tw] OR diabetic renal[tw] OR diabetic glomerul*[tw] OR dialy*[tw] OR hemodia*[tw] OR haemodia*[tw] OR ckd[tw] OR esrd[tw] OR ((diabet*[tw] OR "Disease Progression"[mh:noexp] OR "Recurrence"[mh:noexp]) AND nephropath*[tw]) OR uremi*[tw] OR uraemi*[tw] OR proteinuri*[tw] OR nephrosclerosis[tw] OR glomerulosclerosis[tw] OR glomerular sclerosis[tw] OR "Glomerular Filtration Rate"[majr:noexp] OR microalbuminuri*[tw] OR macroalbuminuri*[tw] OR albuminuri*[tw] OR calciphylaxis[tw] OR secondary hyperparathyroidism[tw] OR "Hyperparathyroidism, Secondary"[mh:noexp] OR tubulointerstitial fibrosis[tw] OR interstitial fibrosis[tw] OR renal fibrosis[tw] OR kidney fibrosis[tw] OR hyperphosphatemia[tw] OR hyperphosphataemia[tw] OR vascular calcification*[tw] OR alport*[tw] OR denys-drash[tw] OR glomerulopathy[tw] OR hypoalbuminemi*[tw] OR hypoalbuminaemi*[tw] OR multicystic kidney*[tw] OR polycystic kidney*[tw] OR cystic kidney*[tw] OR tenckhoff[tiab] OR kidney disease*[tw] OR kidney failur*[tw] OR kidney function*[tw] OR kidney insufficienc*[tw] OR kidney disorder*[tw] OR kidney dysfunction[tw] OR renal replacement[tw] OR renal disease*[tw] OR renal failur*[tw] OR renal function*[tw] OR renal insufficienc*[tw] OR renal disorder*[tw] OR renal dysfunction[tw] OR ((kidney[tw] OR renal[tw]) AND (ckf[tw] OR crd[tw] OR crf[tw] OR eskd[tw] OR eskf[tw] OR esrf[tw] OR hyperparathyroidism[tw] OR end-stage[tw] OR endstage[tw] OR eGFR[tiab])) OR ((kidney transplant*[tiab] OR renal transplant*[tiab]) AND (candidates[tiab] OR wait list*[tiab] OR waiting list*[tiab])) OR ((ureteral obstruction[tw] OR nephritis OR glomerulonephritis OR nephrop* OR (obstruct*[tiab] AND (kidney*[tiab] OR renal[tiab] OR nephropathy[tiab]))) AND (sclerosi*[tw] OR fibrosi*[tw] OR fibrotic[tw])) |
| | Genetic testing | (("genetic test*") OR ("exome sequenc*") OR (WES) OR ("genome sequenc*") OR (WGS) OR ("gene panel*") OR ("targeted panel*") OR ("multigene panel*") OR ("targeted exome*") OR ("targeted gene sequenc*") OR ("genomic testing platforms") OR (next generation sequenc*") OR (NGS) OR ("massively parallel sequenc*") OR (MPS)) |
| Embase | Chronic kidney disease | (chronic kidney or chronic renal or chronic glomerul* or chronic nephro* or progressive kidney or progressive glomerul* or progressive nephro* or diabetic kidney or diabetic renal or diabetic glomerul* or dialy* or hemodia* or haemodia* or ckd or esrd).tw.<br><br>((diabet* or disease progression or recurrence) and nephropath*).tw.<br><br>(uremi* or uraemi* or proteinuri* or nephrosclerosis or glomerulosclerosis or glomerular sclerosis or Glomerular Filtration Rate or microalbuminuri* or macroalbuminuri* or albuminuri* or calciphylaxis or secondary hyperparathyroidism or secondary hyperparathyroidism or tubulointerstitial fibrosis or interstitial fibrosis or renal fibrosis or kidney fibrosis or hyperphosphatemia or hyperphosphataemia or vascular calcification* or alport* or denys-drash or glomerulopathy or |

| | | |
|---|---|---|
| | | hypoalbuminemi* or hypoalbuminaemi* or multicystic kidney* or polycystic kidney* or cystic kidney* or tenckhoff or kidney disease* or kidney failur* or kidney function* or kidney insufficienc* or kidney disorder* or kidney dysfunction or renal replacement or renal disease* or renal failur* or renal function* or renal insufficienc* or renal disorder* or renal dysfunction).tw.<br><br>((kidney or renal) and (ckf or crd or crf or eskd or eskf or esrf or hyperparathyroidism or end-stage or endstage or eGFR)).tw.<br><br>((kidney transplant* or renal transplant*) and (candidates or wait list* or waiting list*)).tw.<br><br>((ureteral obstruction or nephritis or glomerulonephritis or nephrop* or (obstruct* and (kidney* or renal or nephropathy))) and (sclerosi* or fibrosi* or fibrotic)).tw. |
| | Genetic testing | (genetic test or exome sequenc* or WES or genome sequenc* or WGS or gene panel* or targeted panel* or multigene panel* or targeted exome* or targeted gene sequenc* or genomic testing platform* or next generation seqeunc* or NGS or massively parallel sequenc* or MPS).tw. |

Footnote: Filters for date of publication were used (January 1st, 2005, to present – December 31st, 2024). Filter to include only studies done in humans was also used.

**Table S2. Predefined excel template for data extraction including voting results for inclusion as an eligibility criterion for kidney genetic testing**

| Category | INITIAL Consensus Statement/Criteria (July 2023) | Vote | REVISED INTERNAL Consensus Statement/Criteria (October 16, 2023) | REVISED EXTERNAL Consensus Statement/Criteria (July 24, 2024) | FINAL Consensus Statement / Criteria |
|---|---|---|---|---|---|
| Family History | In individuals with >1 first or second degree relative with kidney disease, familial nephropathy and/or hereditary nephritis in the family, renal genetic testing should be initiated. | E: 7<br>DNE: 1<br>RWA: 2 | In individuals with CKD with >1 first or second degree relative with a similar CKD phenotype, genetic assessment could be considered. Individuals with CKD who are diagnosed with familial nephropathy and/or hereditary nephritis, where the exact etiology is not clear, genetic assessment can be considered. | In individuals with CKD with a first- or second-degree relative with a similar CKD phenotype, genetic assessment should be considered.<br>In cases where the clinical presentation is highly suggestive of a monogenic disorder, but family history is unknown e.g., the patient was adopted, genetic assessments should be considered. | In individuals with CKD with a first- or second-degree relative with a similar subtype of CKD, genetic testing should be considered. In cases where the clinical presentation is highly suggestive of a genetic kidney disease, but family history is unknown or negative (e.g., the patient was adopted), genetic assessments should still be considered. |
| Chronic Kidney Disease (CKD) of Unknown Cause | In individuals where they have end-stage kidney disease (ESKD) <50 years with no identifiable cause detectable by renal biopsy, biochemistry, imaging or clinical assessment, renal genetic testing should be initiated. | E: 6<br>DNE: 1<br>RWA: 1 | In individuals who are diagnosed with CKD <35 years and have stage 3 or higher CKD or ESKD onset <50 years, where there is no identifiable cause, genetic testing should be initiated. Outside of these ages, genetic testing could be initiated following expert consultation.<br><br>Any individual with CKD onset diagnosed <18 years without an identifiable cause should consider genetic testing." | NO CHANGE | Chronic Kidney Disease of unknown cause (CKDu) is defined as a decrease in estimated glomerular filtration rate (eGFR) of less than 60 mL/min/1.73 $m^2$ and/or the presence of kidney damage (including hematuria, proteinuria, or structural anomalies of the kidney and/or genitourinary tract) lasting for three months or longer, where no definitive primary kidney disease is identified. This includes individuals with both positive and negative family histories of kidney disease[16]. The diagnostic yield from genetic testing for CKDu ranges from 12-56%.<br>Currently, genetic testing is recommended for individuals diagnosed with CKDu who are ≤35 years old and have stage 3 or higher CKD or ESKD onset at ≤50 years. For individuals outside of these age ranges, genetic testing can still be considered following expert consultation and/or if additional clinical information is available that may impact management.<br>Any individual with CKDu diagnosed at ≤18 years, without an identifiable cause, should be considered for genetic testing. |

| | | | | | |
|---|---|---|---|---|---|
| Age of Onset | If CKD < 35 years and ESKD <50 years; CKD >35 if cause remains unclear after investigation, renal genetic testing should be initiated. | E: 6<br>DNE: 1<br>RWA: 1 | REMOVED – as | | |
| History of Consanguinity | In individuals with history of parental consanguinity, renal genetic testing should be initiated. | E: 3<br>DNE: 3<br>RWA: 4 | In an individual with CKD with an assumed autosomal recessive inheritance and/or history of consanguinity, genetic testing should be considered. | NO CHANGE | NO CHANGE |
| Extra-Renal Involvement | Renal genetic testing should be initiated in CKD plus extra-renal features (examples of features in additional columns, these can be discussed at our next Expert Group meeting. Please suggest any features important to highlight) | E: 7<br>DNE: 0<br>RWA: 3 | In an individual with CKD and features of extra-renal involvement and/or multi-system disease, genetic testing should be considered.<br><br>Examples of extra-renal features and multi-system disease include developmental or functional impairment including learning disabilities, polydactyly, facial dysmorphisms, hearing impairment and/or other ear anomalies, retinal disease, early onset gout preceding the onset of CKD, and features suggestive of maturity onset diabetes of the young (MODY). This is not an exhaustive list but rather examples of extra-renal features of disease. | In an individual with CKD and features of extra-renal involvement and/or multi-system disease, genetic testing should be considered. | |

| | | | | | |
|---|---|---|---|---|---|
| Multi-System Disease | Renal genetic testing should be initiated in CKD in individuals plus multi-system disease (examples of diseases in additional columns, these can be discussed at our next Expert Group meeting. Please suggest any diseases important to highlight). | E: 5<br>DNE: 2<br>RWA: 2 | REMOVED | REMOVED | |
| Cystic Kidney Disease | Consider testing to enable diagnosis if atypical disease, potential alternative diagnosis, negative family history, younger with fewer or smaller cysts, or uncertain / doubt of diagnosis | E: 6<br>DNE: 0<br>RWA: 3 | Consider testing in individuals with cystic kidney disease where there is a suspicion of an alternative diagnosis other than PKD1 and PKD2, for example younger age of onset with fewer or smaller cysts, or where there is uncertainty or doubt of diagnosis.<br><br>Genetic testing could also be considered in individuals with assumed ADPKD where the genetic assessment is anticipated to influence clinical management (i.e., living donation, pre-conception counselling). | 2+ cysts in each kidney on USS at any age1 and at least one of the following:<br><br>• Young age of onset (<18 years of age)<br><br>• Atypical cystic kidney disease or diagnostic uncertainty<br><br>• Results required for clinical management (i.e., living donation, risk assessment)<br><br>• Progressive disease<br><br>• Negative family history of CKD (consider targeted PKHD1 if high clinical suspicion for autosomal recessive PKD) | **Typical Classical Cystic Kidney Disease**: If confirmation of genetic cause of disease is required for clinical management, informing clinical decision making in living kidney donation, at-risk family member(s) and/or to inform treatment decisions.<br><br>**Atypical Cystic Kidney Disease:** If disease is characterized by fewer cysts, asymmetrical cyst distribution, smaller-than-expected kidney size, and/or a negative family history[37]. |

| | | | | | |
|---|---|---|---|---|---|
| PKD testing children | The decision to test for ADPKD in asymptomatic children and young people (CYP) at risk of developing ADPKD, should be undertaken jointly between health professionals and parents and, wherever possible, the young person after genetic counselling. | E: 6<br>DNE: 1<br>RWA: 3 | The decision to test for ADPKD in asymptomatic children and young people at risk should only be undertaken after genetic assessment and counselling. | REMOVED | |
| Haematuria or collagenopathy (>6 months haematuria with no obvious cause) | Consider genetic testing if positive family history OR kidney biopsy features Alport/Thin Basement Membrane Disease (TBMD) OR clinical features of Alport | E: 7<br>DNE: 1<br>RWA: 1 | In individuals with persistent haematuria (>6 months haematuria with no obvious cause) consider genetic testing in the following:<br>o Positive family history of haematuria of unclear etiology OR<br>o Positive family history of Alport Syndrome or Alport Disease<br>o Biopsy features suggestive of Alport/Thin Basement Membrane Disease OR<br>o Clinical features suggestive of Alport syndrome, i.e., haematuria with hearing impairment and/or eye pathology. | Persistent haematuria (>6 months)[2] with no obvious cause *and* at least one of the following:<br>a. Positive family history of haematuria of unknown cause<br>b. Biopsy features suggestive of Alport Syndrome or Alport Disease<br>c. Clinical features suggestive of Alport Syndrome (i.e., hearing impairment and/or eye pathology)<br>OR<br><br>Acute kidney injury associated with thrombocytopenia, microangiopathic hemolytic anemia and negative | Included in the proteinuric kidney disease panel testing |

| | | | | | |
|---|---|---|---|---|---|
| | | | | Coombs test *and* at least one of the following:<br>a. Positive family history<br>b. Required for planning C5 inhibitor therapy<br>c. Required for transplant planning<br><br>If test results are negative can consider a comprehensive approach to testing if clinical suspicion remains high or there are additional risk factors including a positive family history or extra-renal features of disease**.** | |
| Nephronophthisis | Consider genetic testing in CKD with persistent hyperechoic kidneys on renal USS without other obvious cause and/or chronic tubulo-interstitial pattern of injury on kidney biopsy with no obvious cause | E: 8<br>DNE: 0<br>RWA: 0 | In patients with isolated CKD and/or CKD associated with features of suggestive of nephronophthisis or ciliopathies, consider genetic testing if persistent hyperechoic kidneys on renal ultrasound without other obvious cause and/or chronic tubulo-interstitial pattern of injury on kidney biopsy with no obvious cause. | CKD, isolated or associated with features of nephronophthisis or ciliopathies *and* at least one of the following:<br>a. Persistent unexplained echogenic kidney on imaging<br>b. Chronic tubulo-interstitial pattern of injury on kidney biopsy | Included in the comprehensive approach to testing guidelines due to non-specific clinical presentation |
| *HNF1B* testing/ Renal Cysts and Diabetes Syndrome | Consider genetic testing for renal cysts, other structural renal and/or genital tract anomalies, electrolyte | E: 6<br>DNE: 1<br>RWA: 2 | In patients with high index of clinical suspicion for *HNF1B* related disease/ renal cysts and diabetes syndrome, consider specific gene testing. | | |

| | | | | | |
|---|---|---|---|---|---|
| | abnormalities, abnormal liver function, early-onset diabetes mellitus and gout. If features of renal cysts and diabetes syndrome present consider microarray, and if negative consider sequencing HNF1B. | | | | |
| CAKUT testing | Consider genetic testing if:<br>1. 1st degree relative with CAKUT OR<br>2. unexplained end-stage renal disease OR<br>3. CKD stage >2 OR<br>4. Extrarenal features/ syndromic | E: 5<br>DNE: 1<br>RWA: 3 | Consider genetic testing in patients diagnosed with CAKUT if the following criteria are met:<br>• Positive family history with ≥ 1st degree relative with CAKUT OR;<br>• Positive family history of unexplained kidney disease OR;<br>• Affected individual has impaired kidney function with CKD stage >2 OR;<br>• Presence of extrarenal features or multisystem disease. | Abnormality in structure, function, size, shape, or position of the kidney and/or genitourinary tract *and* at least one of the following:<br>• Positive family history of CAKUT or unexplained kidney disease<br>• Impaired kidney function with CKD stage >2<br>• Presence of extra- renal features or multisystem disease<br>If there is evidence of multi-system disease, consider a comprehensive approach including either exome or genome approach as first line of testing. | Abnormalities in the structure, function, size, shape, or position of the kidney and/or genitourinary tract *and* at least one of the following:<br>• **Family History**: Positive family history of CAKUT or unexplained kidney disease.<br>• **Impaired Kidney Function**: CKD stage ≥2.<br>• **Extra-Renal or Multisystem Disease**: Presence of extra-renal features or multisystem involvement.<br>**Additional Consideration**: For childhood-onset disease with multi-system disease, CNV (copy number variant) analysis using microarray may be considered prior to panel testing, given the high rate of structural variants associated with disease[36]. If there is evidence of multisystem disease, consider a comprehensive genome wide sequencing approach as the first-line test. |
| Proteinuric renal disease, Glomerulopathy, podocytopathy | Consider testing if presence of:<br>1. Steroid-resistant nephrotic syndrome presenting at any age, OR | E: 8<br>DNE: 0<br>RWA: 0 | No changes | Disease of the renal glomerulus characterized by any one of the following:<br>a. Steroid-resistant nephrotic syndrome at any age | **Steroid-Resistant Nephrotic Syndrome** at any age.<br>**Proteinuria with Focal Segmental Glomulo-Sclerosis (FSGS) or Diffuse Mesangial Sclerosis (DMS)** on kidney biopsy, with any of the following features:<br>○ Positive family history of kidney disease.<br>○ Unclear cause of the proteinuria. |

| | | | | | |
|---|---|---|---|---|---|
| | 2. Proteinuria with a histological picture of focal segmental glomerulosclerosis (FSGS) or diffuse mesangial sclerosis (DMS) on biopsy, with no identifiable cause, where a transplant or immunosuppression is planned | | | b. Proteinuria with focal segmental glomerulosclerosis (FSGS) or diffuse mesangial sclerosis (DMS) on biopsy, and any of the following features: positive family history of CKD or ESKD OR where the cause is unclear OR a kidney transplant is planned<br>c. Any glomerulonephritis of uncertain etiology onset <35 years old | o A kidney transplant is planned and genetic testing will inform post-transplant risk of recurrence.<br>**Glomerulonephritis of Uncertain Etiology** with clinical suspicion for a genetic cause.<br><br>**Persistent Hematuria (>6 months)**[1] with no obvious cause, and one of the following:<br>o Positive family history of hematuria<br>o Biopsy features suggestive of Alport Syndrome or Alport Disease.<br>o Clinical features suggestive of Alport Syndrome (e.g., hearing impairment and/or eye pathology). |
| Tubulopathies or persistent metabolic abnormalities | Patients with a primary renal tubulopathy presenting as one of the following conditions should be considered for genetic testing:<br>1. Hypokalaemic alkalosis with normal or low blood pressure (e.g. Bartter/Gitelman syndromes), OR<br>2. Hypokalaemic alkalosis with elevated blood pressure (e.g. Liddle syndrome), OR<br>3. Hyperkalaemic | E: 7<br>DNE: 0<br>RWA: 1 | No changes | Patients with a primary kidney tubulopathy can be considered for genetic testing after full tubular phenotyping is undertaken (i.e., 24 hr urine and blood test) and presenting with any of the following conditions:<br><br>1. Hypokalaemic alkalosis plus either:<br>• Normal or low blood pressure suggestive of Bartter/Gitelman syndromes, OR<br>• Elevated blood pressure suggestive of Liddle syndrome, OR<br>2. Hyperkalaemic acidosis plus either:<br>• Low/normal BP suggestive of PHA type 1, OR<br>• Elevated BP suggestive of PHA type 2, OR | **Suspected Tubulopathies** after full clinical and biochemical phenotyping, and exclusion of secondary causes, consider genetic testing in patients suspected of having any of the following conditions: Dent Disease, Renal Tubular Acidosis, Bartter Syndrome, Gitelman Syndrome, Hypomagnesemia, Pseudohypoaldosteronism, Hypophosphatemia and/or Diabetes Insipidus. For other rare tubulopathies, expert consultation is recommended.<br><br>**Radiologically confirmed nephrocalcinosis** after excluding acquired causes.<br><br>**Recurrent Nephrolithiasis** when a genetic etiology is suspected, particularly with a positive family history or pediatric onset. |

| | | | | | |
|---|---|---|---|---|---|
| | acidosis with low/normal BP (PHA type 1), OR<br>4. Hyperkalaemic acidosis with elevated BP (PHA type 2), OR<br>5. Hypokalaemic acidosis (pRTA and renal Fanconi syndromes), OR<br>6. Hypomagnesaemia, OR<br>7. Nephrogenic diabetes insipidus, OR<br>8. Other rare types of renal tubulopathy seen in an expert center | | | 3. Hypokalaemic acidosis suggestive of pRTA and kidney Fanconi syndromes, OR<br>4. Hypomagnesaemia when other potential etiologies have been excluded (i.e., medication related) OR<br>5. Nephrogenic diabetes insipidus<br><br>For other rare types of kidney tubulopathy, an expert consultation is suggested | |
| Complement mediated kidney disease or MPGN | Consider genetic testing in complement mediated disease if there is:<br>1. Positive family history, AND/OR<br>2. Renal Transplant considered AND/OR<br>3. Commencing or ceasing treatment | E: 8<br>DNE: 0<br>RWA: 1 | In individuals with biopsy proven MPGN or C3GN, consider genetic testing in the following:<br>1. Positive family history, AND/OR<br>2. Renal transplant is considered AND/OR<br>3. Commencing or ceasing treatment | Unexplained membranoproliferative glomerulonephritis (MPGN) that is biopsy proven or complement mediated glomerulonephritis *and* at least one of the following:<br>a. Positive family history<br>b. Diagnosis required for transplant/treatment planning | **Complement Mediated Kidney Disease and Atypical Hemolytic Uremic Syndrome (aHUS) Panel**<br>Unexplained membranoproliferative glomerulonephritis (MPGN) that is biopsy proven or complement mediated glomerulonephritis *and* at least one of the following:<br>• Positive family history.<br>• Diagnosis required for transplant/treatment planning.<br><br>OR<br><br>Clinical diagnosis of aHUS characterized by acute kidney injury associated with thrombocytopenia, microangiopathic |

| | | | | | |
|---|---|---|---|---|---|
| | | | | | hemolytic anemia and negative Coombs test *and* at least one of the following:<br>○ Positive family history.<br>○ Required for planning C5 inhibitor therapy.<br>○ Required for transplant planning. |
| Atypical Hemolytic uremic syndrome | Consider genetic testing if positive family history OR if considering C5 inhibitor therapy (either commencing or discontinuation) OR prior to transplantation. | E: 5<br>DNE: 0<br>RWA: 4 | In patients with suspected atypical hemolytic uremic syndrome (defined as the presence of acute kidney injury, thrombocytopenia, MAHA and Coombs test negative), consider genetic testing in the following:<br>1. Positive family history AND/OR<br>2. If considering C5 inhibitor therapy (either commencing or discontinuation) AND/OR<br>Prior to kidney transplantation | COMBINED WITH COMPLEMENT | COMBINED WITH COMPLEMENT |
| TKD or CKD with bland urine if other causes excluded | Consider genetic testing if 1. Renal impairment caused by tubulointerstitial fibrosis with no glomerular lesion, with no identifiable cause, often associated with medullary cysts, hyperuricaemia or gout, AND<br>2. A first degree relative with TIKD or unexplained end- | E: 8<br>DNE: 0<br>RWA: 0 | In an individual with suspected tubulointerstitial kidney disease defined as CKD with bland urine if other causes excluded, consider genetic testing if:<br>· Kidney biopsy shows a tubulointerstitial pattern of injury or fibrosis with no glomerular lesion, with there are no other identifiable causes, AND/ OR<br>· Presence of hyperuricaemia or gout if young age of onset and preceding the onset of CKD, AND/ OR | In individuals with suspected tubulointerstitial kidney disease defined as CKD with a bland urine if other causes excluded, consider genetic testing if:<br><br>Presence of CKD characterized by tubular damage and interstitial fibrosis in the absence of glomerular lesions with other causes excluded by a bland urine sediment *and* at least one of the following:<br>a. Personal or family history of hyperuricaemia or gout if | |

| | | | | | |
|---|---|---|---|---|---|
| | stage renal disease. Exceptions may be made for patients where the clinical presentation is highly suggestive of a monogenic aetiology, but family history is unknown eg. the patient was adopted | | · Positive family history of TIKD or unexplained end-stage renal disease.<br>Exceptions may be made for patients where the clinical presentation is highly suggestive of a monogenic TIKD, but family history is unknown e.g. the patient was adopted | young age of onset and preceding the onset of CKD<br>b. Positive family history of TKD or unexplained CKD<br><br>Exceptions may be made for patients where the clinical presentation is highly suggestive of a monogenic TKD, but family history is unknown e.g., the patient was adopted.<br><br>*MUC1* kidney disease should be considered in individuals with adult-onset CKD, with a bland urine and a positive family history of CKD in an autosomal dominant pattern of inheritance.<br><br>**Note: MUC1* testing is not currently available on any commercially available sequencing platform. If ADTKD due to a *MUC1* mutations is suspected, please seek renal genetic expert advice prior to proceeding with testing. | |
| Amyloidosis | Consider genetic testing if clinical features suggestive of hereditary amyloidosis which may include restrictive cardiomyopathy, | E: 9<br>DNE: 0<br>RWA: 1 | In individuals with biopsy proven amyloidosis, consider genetic testing if clinical features suggestive of hereditary amyloidosis which may include restrictive cardiomyopathy, autonomic and peripheral neuropathy, renal impairment, | Presence of abnormal amyloid deposits in the kidney *and* but not limited to one of the following clinical features suggestive of hereditary amyloidosis:<br>a. Restrictive cardiomyopathy<br>b. Autonomic and peripheral neuropathy | Presence of abnormal amyloid deposits in the kidney *and* clinical features suggestive of hereditary amyloidosis when other secondary causes of amyloidosis have been ruled out. Clinical features include but not limited to:<br>• Restrictive cardiomyopathy<br>• Autonomic and peripheral neuropathy<br>• Gastrointestinal involvement<br>• Positive family history of amyloidosis |

| | | | | | |
|---|---|---|---|---|---|
| | autonomic and peripheral neuropathy, renal impairment, or gastro-intestinal symptoms | | or GI symptoms AND/OR a positive family history of amyloidosis | c. Kidney impairment<br>d. Gastrointestinal involvement<br>e. Positive family history of amyloidosis | |
| Nephrocalcinosis or nephrolithiasis | Consider genetic testing when acquired causes have been excluded<br>• Where a primary endocrine disturbance of calcium homeostasis is identified, the appropriate specific test<br>should be used<br>• In individuals with an identifiable primary renal disorder, the specific test for that disorder should be used where genetic testing is appropriate<br>• Individuals with nephrocalcinosis likely to be caused by Bartter syndrome can be tested using this<br>indication; | E: 5<br>DNE: 2<br>RWA: 0 | Consider genetic testing in individuals with recurrent nephrolithiasis or radiological imaging confirmed nephrocalcinosis, only when acquired causes have been excluded and a primary endocrine or renal disorder is suspected.<br>Patients with recurrent nephrolithiasis and a positive family history should only undergo genetic testing after expert consultation.<br>Individuals with nephrocalcinosis and suspected tubulopathy (Bartter syndrome) consider genetic testing as per renal tubulopathies guidelines. | Recurrent nephrolithiasis or radiologically confirmed nephrocalcinosis, acquired causes excluded, and one of the following:<br>**a.** A primary endocrine disturbance or a primary kidney disorder is suspected.<br>b. Patients with recurrent nephrolithiasis and a positive family history should only undergo genetic testing after expert consultation and when other primary causes have been excluded. | |

| | | | | | |
|---|---|---|---|---|---|
| | individuals with a different presentation of Bartter syndrome should be tested using R198 Renal tubulopathies | | | | |
| Suspicion of specific gene | In individuals where a specific gene associated with kidney disease is suspected, consider genetic testing for the gene in question. For example, individuals with a specific matching (biochemical) phenotype (Alport, Fabry, Specific tubulopathy, Amyloidosis), genetic testing is always indicated. | E: 8<br>DNE: 1<br>RWA: 1 | NO CHANGE | In individuals with CKD where a specific genetic condition is suspected, proceed with genetic testing for the gene(s) in question. | This is reserved for individuals with CKD where there is a very high index of clinical suspicion for a specific genetic condition. In these cases, clinicians can proceed with genetic testing for the gene(s) in question. If the specific genetic condition in question is not clear, the Disease-Specific Panel or Comprehensive Approach to testing may be more appropriate |
| Reproductive planning | In individuals looking for pre-conception counselling or pre-implantation counselling, genetic testing for renal conditions should be initiated | E: 5<br>DNE: 2<br>RWA: 3 | In individuals with CKD and/or with personal of family history of kidney disease looking for pre-conception counselling or pre-implantation counselling, genetic testing for renal conditions should be initiated. | In individuals with CKD and/or with family history of kidney disease looking for reproductive planning, genetic testing for kidney conditions could be offered after expert consultation. | For individuals with CKD or a family history of kidney disease who are planning for reproduction, genetic testing may be offered to assess potential genetic risks in offspring. Expert consultation is recommended to help guide decision-making, ensuring appropriate counseling and informed reproductive choices. |
| At-risk relatives in families with | If pathogenic/ likely pathogenic variant in | E: 7<br>DNE: 0 | If pathogenic/ likely pathogenic variant in a gene associated with | RENAMED TO CASCADE TESTING: If pathogenic/likely pathogenic variant | When a pathogenic or likely pathogenic variant is confirmed in an individual, cascade testing can be considered for at-risk |

| | | | | | |
|---|---|---|---|---|---|
| monogenic kidney disease | a gene associated with renal condition has been confirmed in an individual following genetic testing, cascade testing should be offered to all first-degree relatives for known familial variants | RWA: 3 | renal condition has been confirmed, or presence of VUS if the phenotype presentation is classic (e.g., ADPKD) in an individual following genetic testing, cascade testing should be considered to all first-degree relatives for known familial variants. | in a gene associated with kidney condition has been confirmed, cascade testing for known familial variants should be considered for at-risk family members. If there is a suspicious variant of uncertain (or unknown) significance (VUS) in an affected family member that is consistent with the observed phenotype, family testing can be considered after expert consultation. | family members. If testing has already been performed in a biologically related family member, every effort should be made to obtain the testing report to confirm the familial variant of interest. |
| Potential of recurrence or clinical implication post kidney transplant | Genetic confirmation should be initiated when there is potential or recurrence or clinical implication post kidney transplant. Examples include but not limited to genetic testing to assess for recurrence of FSGS post-transplant or confirmation of Alport Syndrome pre transplant | E: 9<br>DNE: 1<br>RWA: 0 | In individuals proceeding with kidney transplant, consider genetic testing in the following situations:<br>1. ESKD onset <50 year where the cause of CKD is unknown OR<br>2. A specific genetic condition or a heritable cause of kidney disease is suspected OR<br>3.Confirmation of a genetic diagnosis may have clinical implications post-transplant e.g., confirmation of a genetic of FSGS to facilitate counselling on recurrence risk or Alport syndrome testing pre-transplantation OR<br>4. Risk assessment in a biologically related donor | In individuals proceeding with kidney transplant, consider genetic testing in any of the following situations:<br>• ESKD onset <50 year where the cause of CKD is unknown.<br>• A genetic condition or a heritable cause of kidney disease is suspected OR<br>• Confirmation of a genetic diagnosis may have clinical implications post-transplant e.g., confirmation of a genetic FSGS to facilitate counselling on recurrence risk or Alport syndrome testing pre-transplantation Risk assessment in a biologically related donor. | For individuals undergoing kidney transplantation, genetic testing should be considered in the following scenarios:<br>• **ESKD Onset Before Age 50** especially when the cause of CKD is unknown.<br>• **Suspected Genetic Condition**: If a heritable kidney disease is suspected, genetic testing should be considered to inform transplant planning.<br>• **Clinical Implications Post-Transplant**: Pre-transplant genetic testing in individuals in whom a confirmed genetic diagnosis may inform the risk of disease recurrence post-transplant. |
| Impact on living donation | Considering genetic testing to guide selection of potential biologically | E: 9<br>DNE: 0<br>RWA: 1 | NO CHANGE | NO CHANGE | Genetic testing should be considered in the affected individual (the person with CKD who requires a kidney transplant) to confirm specific mutations followed by specific mutation confirmation in unaffected family members. This can help |

| | | | | | |
|---|---|---|---|---|---|
| | related kidney donors | | | | guide the selection of biologically related living kidney donors. Comprehensive/ non-targeted testing in *unaffected* family members wishing to proceed with living kidney donation, is not currently recommended. |
| Treatment implications | Consider genetic testing if and when new treatments become available although this is not recommended as a sole criteria by PGP (see sheet titled "Treatment Implication") | E: 5<br>DNE: 1<br>RWA: 0 | REMOVED | REMOVED | Genetic testing may have important treatment implications. For example, identifying a genetic cause of CKD can inform treatment strategies, potential therapeutic interventions, and kidney transplant planning[17].Specific conditions, such as Focal Segmental Glomerulosclerosis (FSGS) or Alport syndrome, may influence how patients are managed both before and after transplant[42]. Genetic testing should be considered in any individual with CKD in whom confirmation of a genetic diagnosis may have treatment implications. |
| Resistance to treatment | Consider genetic testing if diagnosis will impact on treatment decision - for example steroid resistant nephrotic syndrome or atypical presentation not responding to routine therapy | E: 9<br>DNE: 0<br>RWA: 1 | REMOVED | REMOVED | |
| Semi-rapid testing or need for rapid diagnosis | Consider genetic testing when: 1. Acutely unwell children or adults where monogenic kidney disease is considered highly likely to be the primary cause of the phenotype, OR 2. | E: 6<br>DNE: 1<br>RWA: 3 | NO CHANGE | NO CHANGE | Genetic testing should be considered when a rapid diagnosis is needed, particularly for:<br>• **Acutely Unwell Individuals**: In children or adults where monogenic kidney disease is suspected as the primary cause of disease, rapid genetic testing should be considered to confirm the diagnosis and guide urgent clinical decisions.<br>• **Rapid Changes in Management**: If genetic testing will lead to an immediate change in treatment, such as informing decisions about kidney transplant, |

| | | | | | |
|---|---|---|---|---|---|
| | Where testing will provide an immediate change to treatment or clinical management for the patient e.g. To inform a decision about renal transplant, therapeutic intervention, or prenatal testing for an ongoing at risk pregnancy. | | | | therapeutic intervention or prenatal testing for an at-risk pregnancy, testing should be performed as part of the clinical decision-making process. |
| Neonatal screening/ Multiple congenital anomalies | Consider genetic testing in neonatal population with multiple congenital anomalies including kidney anomalies | E: 9<br>DNE: 0<br>RWA: 1 | No change | | Genome wide sequencing testing should be considered in neonates presenting with multiple congenital anomalies including kidney anomalies consistent with the diagnosis of CAKUT. Early genetic testing can help identify the underlying genetic conditions that may guide treatment and management. |
| CAKUT with normal kidney function | Do not consider genetic testing by nephrologist for CAKUT with normal kidney function, however genetic testing could be considered following a renal genetic consultation | E: 6<br>DNE: 0<br>RWA: 2 | Removed | | |
| Previous genetic diagnosis | If prior genetic testing in individual or family obtain and review for possible | E: 7<br>DNE: 0<br>RWA: 2 | Removed | | |

| | | | | | |
|---|---|---|---|---|---|
| | specific mutation confirmation | | | | |
| Steroid sensitive nephrotic syndrome | Do not consider genetic testing by nephrologist in presence of steroid sensitive nephrotic syndrome unless known monogenic causes become evident over time | E: 8<br>DNE: 0<br>RWA: 0 | Removed | | |
| Immunological cause of ESKD | Do not consider genetic testing by nephrologist if immunological cause of ESKD unless known monogenic causes become evident over time | E: 6<br>DNE: 2<br>RWA: 0 | Removed | | |
| Testing family members for variants of uncertain significance (VUS) | | | | | If a Variant of Uncertain Significance (VUS) is identified in a gene associated with a heritable kidney condition, the VUS should be reviewed by a genetic professional or clinician with expertise in variant interpretation. Testing biologically related relatives for a VUS is generally not recommended. In some cases, testing other affected family members may assist in variant interpretation by determining if a VUS segregates with disease in the family. Please note however, that segregation analysis alone is generally not sufficient evidence to prove pathogenicity. If over time, there is evidence suggesting that the VUS interpretation may have changed, a formal re-interpretation should be requested from the laboratory[13]. In some circumstances, testing unaffected parents may be appropriate to determine whether the variant has been inherited *de novo* and/or in the case of an autosomal recessive |

| | | | | | |
|---|---|---|---|---|---|
| | | | | | condition where a compound heterozygous variant is identified, to determine if variants are inherited *in trans* or *in cis*. In these circumstances, testing in an unaffected family member may be indicated. |
| Criteria for Genome-Wide Sequencing (WGS) | | | | | Genome-Wide Sequencing (WGS) encompasses both exome and genome sequencing and can be considered in the following circumstances as the initial genetic testing approach:<br>**A. Dual Diagnosis Suspected:** If there is suspicion of a non-kidney disease phenotype, in addition to CKD, WGS should be considered.<br>**B. Presence of Multisystem Disease:** In cases where CKD is accompanied by multisystem involvement, WGS should be considered.<br>**C. Cost-Effectiveness considerations:** If ≥2 disease specific gene panels are indicated, WGS may be the more cost-effective and efficient approach to testing. |

BP; blood pressure, CKD; chronic kidney disease, DMS; Diffuse Mesangial Sclerosis, DNE; Do not Endorse, ESKD, end-stage kidney disease, E; Endorse, FSGS; Focal Segmental Glomerulo-Sclerosis, MAHA; microangiopathic hemolytic anemia, PHA, pseudo hypoaldosteronism; pRTA, proximal Renal Tubular Acidosis, RWA, Recommended with Adaptations

Table S3. Environmental Scan of Currently Available Genetics Testing Panels for Patients with Chronic Kidney Disease as of April 2023.

| Lab | Test Name | Genes |
|---|---|---|
| LHSC | Amyloidosis | *TTR* |
| KGH | Amyloidosis | *TTR* |
| UHN | Amyloidosis | *APOA1, APOA2, B2M, FGA, GSN, LYZ, TTR* |
| LHSC | Cystinosis | *CTNS* |
| MSH | Autosomal dominant PKD Analysis | *PKD1, PKD2* |
| MSH | Autosomal recessive PKD Analysis | *PKHD1* |
| MSH | PKD Full Analysis | *PKD1, PKD2, PKHD1* |
| MSH | PKD1 Deletion/Duplication only | *PKD1* |
| MSH | PKD1 Sequencing only | *PKD1* |
| MSH | PKD2 Deletion/Duplication only | *PKD2* |
| MSH | PKD2 Sequencing only | *PKD2* |
| MSH | PKHD1 Deletion/Duplication only | *PKHD1* |
| MSH | PKHD1 Sequencing only | *PKHD1* |
| HSC | Renal Disease: atypical Hemolytic Uremic Syndrome / C3 glomerulonephritis (aHUS/C3G) | *C3, CD46, CFB, CFH, CFHR1, CFHR2, CFHR3, CFHR4, CFHR5, CFI, DGKE, THBD* |
| HSC | Renal Disease: Focal Segmental Glomerulonephritis Syndrome (FSGS) and membranous nephropathies | *ACTN4, ADCK4, CD2AP, COQ2, INF2, LAMB2, LMX1B, MYH9, NPHS1, NPHS2, PDSS2, PLCE1, SCARB2, SMARCAL1, TRPC6, WT1* |
| HSC | Hereditary Hearing Loss: Syndromic Hearing Loss (Alport Genes) | *COL4A3, COL4A4, COL4A5* |
| HSC | Fabry Disease | *GLA* |

aHUS; atypical hemolytic uremic syndrome, C3G; C3 glomerulonephritis, PKD; polycystic kidney disease, FSGS; Focal Segmental Glomerulosclerosis, HSC; The Hospital for Sick Children, LHSC; London Health Sciences Centre, KGH; Kingston Health Sciences Centre, UHN; University Hospital Network Toronto, MSH; Mount Sinai Hospital

Table S4. Genome-wide Sequencing Ontario Eligibility Criteria for Clinical Exome Sequencing.

| General Criteria | Specific criteria |
|---|---|
| Clinical presentation (must meet ≥ 2 items) | 1. Moderate to severe developmental or functional impairment<br>2. Multisystem involvement<br>3. Progressive clinical course<br>4. Differential diagnosis includes ≥ 2 well defined conditions requiring evaluation by multiple targeted gene panels.<br>5. Suspected severe genetic syndrome NYD for which multiple family members are also affected, or where parents are consanguineous. |
| Management Impact (must meet ≥ 1 item) | 6. Will limit further invasive diagnostic investigations.<br>7. Results allow for specific and informed reproductive decision making (for patient or parents)<br>8. Will enable identification of at-risk family members and facilitate early intervention. |

**Legend:** NYD; not yet defined. See Patient Eligibility - Genome-wide Sequencing Ontario (gsontario.ca)

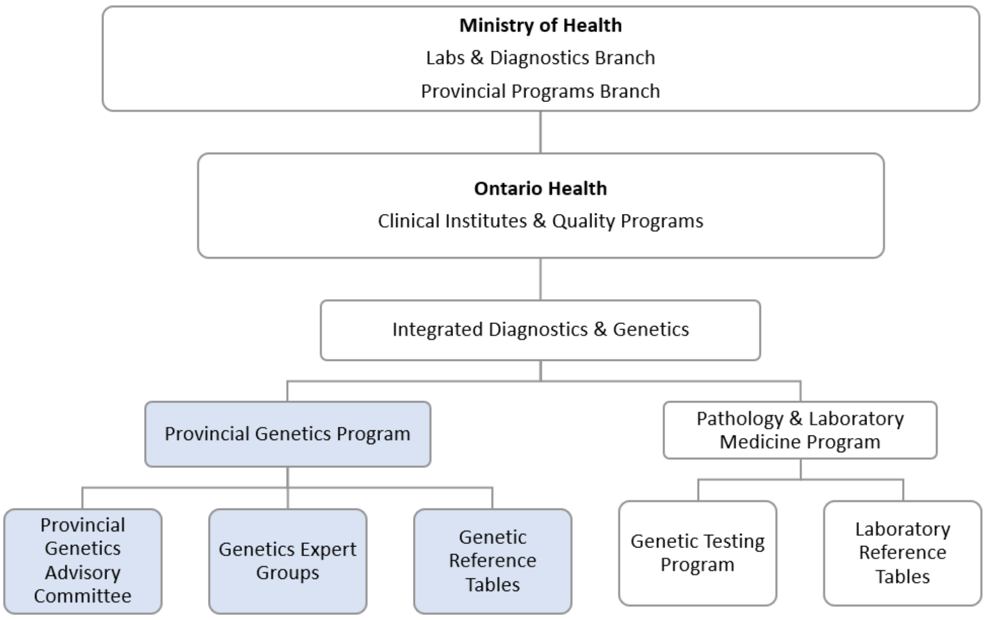


Figure S1. Ontario Health Provincial Genetics Program (PGP) Governance Structure

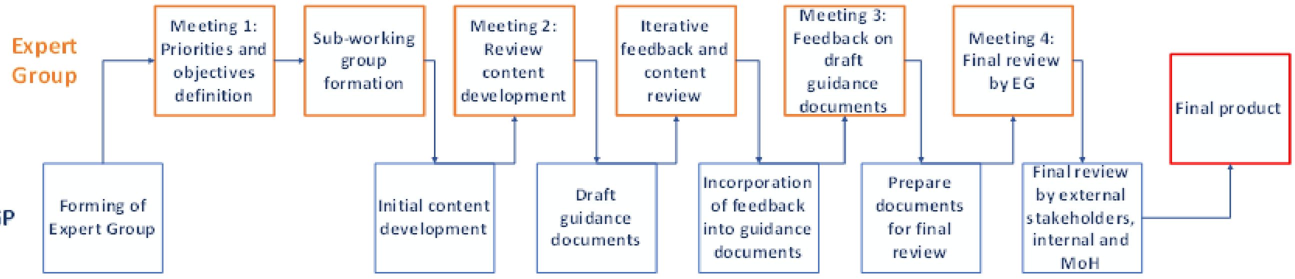


Figure S2. Ontario Health Provincial Genetics Program (PGP) Workflow and Process Map for each Expert Group.
Legend: EG; Expert Group, MoH; Ministry of Health
